# Impact of sialyltransferase deletion in mature T cells on control of subcutaneous and metastatic cancers

**DOI:** 10.1093/glycob/cwag065

**Published:** 2026-08-19

**Authors:** Xiaoshuang Wang, James C Paulson

**Affiliations:** The School of Medicine, Nankai University, 94 Weijin Road, Tianjin 300071, China; Department of Immunology and Microbiology, The Scripps Research Institute, 10555 N. Torrey Pines Road, La Jolla, CA 92037, United States; Department of Immunology and Microbiology, The Scripps Research Institute, 10555 N. Torrey Pines Road, La Jolla, CA 92037, United States

**Keywords:** CD8^+^ T cell, metastasis, sialic acid, sialyltransferases, tumor

## Abstract

To investigate the roles of the α2,3-sialyltransferase ST3Gal1 and the α2,6-sialyltransferase ST6Gal1 in T cell-mediated tumor control, we generated mice with mature T cell-specific deletion of ST3Gal1 (T-ST3KO) or ST6Gal1 (T-ST6KO) using distal Lck-Cre-mediated recombination. Deletion of ST3Gal1 in mature T cells did not affect thymic T cell development but resulted in a reduction of peripheral CD8^+^ T cells. In contrast, ST6Gal1 deletion had minimal impact on T cell development and peripheral T cell abundance. Following in vitro stimulation of isolated T cells from T-ST3KO and parental wild type (WT) mice with anti-CD3 plus anti-CD28/CD80-Fc, CD8^+^ T cells from T-ST3KO mice exhibited enhanced activation and an increased frequency of CD44 positive memory-like T cells, while comparison of T cells from T-ST6KO and the parental WT mice showed no significant changes. Despite the activated CD8^+^ T cell phenotype in T-ST3KO mice, subcutaneous MC38 tumors displayed accelerated growth. In contrast, tumor progression in T-ST6KO mice was unchanged from the parental WT mice. Notably, T-ST6KO mice developed increased pulmonary metastases following intravenous challenge with B16F10 melanoma cells, whereas metastatic burden was unaffected in T-ST3KO mice. These findings demonstrate distinct and non-redundant roles for ST3Gal1- and ST6Gal1-mediated sialylation in regulating T cell function and antitumor immunity and reveal context-dependent effects of T cell intrinsic sialylation in controlling primary tumor growth and metastatic dissemination.

## 1. Introduction

Terminal sialic acid (Sia) residues that cap N- and O-linked glycans on cell-surface glycoproteins play critical roles in immune-cell regulation of cancers (Varki and Gagneux 2012; Pearce and Laubli 2016; Edgar et al. 2021; Bashian et al. 2026). Aberrant hypersialylation on cancer cells creates a suppressive immune environment by shielding cell-to-cell contact and creating ligands for **S**ialic acid **I**mmuno**g**lobulin **lec**tins, Siglecs, which are immune checkpoints that dampen anti-tumor immunity (Crocker et al. 2007; Macauley et al. 2014; Boligan et al. 2015; Coccimiglio et al. 2024; Yang et al. 2024). Reducing sialylation in the tumor microenvironment using inhibitors of sialyltransferases (STs) or sialidases (Bull et al. 2018; Gray et al. 2020; Stanczak et al. 2022; Kasahara et al. 2024; Tsai et al. 2024), or blocking Siglec signaling with antibody or small molecule antagonists (Wen et al. 2024; Xiao et al. 2024; Zhao et al. 2024), have shown great promise for enhancing tumor control as a pharmacological strategy against cancer.

Although reducing hypersialylation of cancer cells to stimulate immune responses has been the primary motivation to target the Sia/Siglec axis (Gray et al. 2020; Laubli and Varki 2025; Bashian et al. 2026), complete removal of sialic acids in MC38 tumors by deletion of the cytidine monophosphate N-acetylneuraminic acid synthetase gene (*Cmas*), required for Sia synthesis, promotes more rapid tumor growth of the tumors (Cornelissen et al. 2019). Such observations show that the immune responses involving the Sia/Siglec axis in tumor control are complex, and that a deeper understanding of the mechanisms involved will be required for development of improved therapies (Laubli and Varki 2025; O'Neill et al. 2025; Ray et al. 2025; Bashian et al. 2026). In this regard, for strategies targeting the Sia/Siglec axis that reduce sialylation of both cancer cells and immune cells in the tumor microenvironment, it is important to understand the impact of the loss of Sia on all cells that drive immune responses.

It is well documented that CD8^+^ T cells are a key cellular mediator of enhanced tumor control when targeting the Sia/Siglec axis (Laubli and Varki 2025; Bashian et al. 2026). Indeed, injection of the sialyltransferase inhibitor 3Fax-Neu5Ac into melanoma tumors caused tumor dramatic regression driven primarily by CD8^+^ T cells (Bull et al. 2018). Similarly, suppression of HER2 positive breast cancer using an anti-HER2-sialidase conjugate was found to be dependent on CD8^+^ T cells, and was also dependent on Siglec-E despite the fact that there was no expression of Siglec-E on T cells (Gray et al. 2020). Direct targeting of T cells using a sialidase-anti-murine programmed cell death protein-1 (PD-1) antibody fusion (Garabedian et al. 2025), or a sialidase tethered to a bi-specific tumor-T cell engager (Yang et al. 2024), also enhanced T cell-mediated tumor cell killing. Because of the importance of CD8^+^ T cells in mediating anti-cancer immune responses, we set out to determine if genetic deletion of key sialyltransferases would impact T cell-mediated immunity in a tumor setting.

Two key sialyltransferases, ST3Gal1 and ST6Gal1, are of particular interest because their expression is variably regulated during T cell development and activation. The use of peanut agglutinin (PNA) to distinguish immature (PNA^+^) and mature (PNA^−^) T cells in thymus tissue sections is well documented to reflect changes in the expression of ST3Gal1 (Gillespie et al. 1993). PNA recognizes the non-sialylated T antigen (Galβ1-3GalNAcαThr/Ser) present on surface glycoproteins of immature T cells. The mature T cells become PNA^−^ as a result of the upregulation of the sialyltransferase ST3Gal1 that converts T-antigen to sialylated T antigen (Siaα2–3Galβ1-3GalαThr/Ser) (Gillespie et al. 1993; Baum et al. 1996; Fukuda and Tsuboi 1999; Priatel et al. 2000; Comelli et al. 2006). Upon activation of naïve T cells, expression of ST3Gal1 is downregulated, resulting in conversion back to PNA^+^ (Priatel et al. 2000; Amado et al. 2004).

In mice, ST6Gal1 is the dominant sialyltransferase for attaching sialic acids to N-linked glycans on T cells in the sequence Siaα2–6Galβ1–4GlcNAc (Comelli et al. 2006). ST6Gal1 is differentially expressed in early CD4/CD8 double negative stage 1–4 (DN1–4) T cell precursors, with high expression in DN1, low expression in DN2-DN3, and re-expression in DN4 precursors and in mature single positive CD4 and CD8 precursors, coinciding with the expression of the T cell receptor (Marino et al. 2008). Activation of mature naïve T cells in vitro results in dramatic downregulation of ST6Gal1 in CD4^+^ and CD8^+^ T cells, with concomitant loss of α2–6 sialic acids on N-linked glycans (Comelli et al. 2006; Izzati et al. 2025). Activation of CD8^+^ T cells in vivo during infection with lymphocytic choriomeningitis virus (LCMV) or *Toxoplasma gondii* similarly results down regulation of ST6Gal1 as inferred from in loss of NeuAcα2–6Gal terminated cell surface sialosides detected with *Sambucus nigra agglutinin* (SNA) (Sierra-Ulloa et al. 2024; Izzati et al. 2025). Importantly, the down regulation of ST6Gal1 in T cells is asymmetric, with is greater downregulation in activated CD8^+^ T cells than CD4^+^ T cells, and in CD4^+^ subsets there is down regulation in Th1 and Th17 cells, but minimal downregulation in Th1 cells (Toscano et al. 2007; Yu et al. 2023). Thus, ST6Gal1 is variably regulated in T cells during development, activation and differentiation.

In addition to their regulated expression, ST3Gal1 and ST6Gal1 produce the ligands of Siglec E (ST3Gal1) and CD22/Siglec-2 (ST6Gal1), which are expressed respectively on myeloid cells and B cells that engage T cells for antigen presentation (Crocker et al. 2007; Lin et al. 2025). Thus, these two sialyltransferases are of interest for their potential to regulate immune responses in biological contexts relevant to the tumor microenvironment.

Mice with systemic or T cell-specific deletion of the ST3Gal1 and ST6Gal1 genes have provided insights into the roles of these enzymes and their products on T cell development and T cell-mediated immune responses (Irons et al. 2024). Mouse lines have been developed with a systemic ST3Gal1 knockout, and a T cell specific knockout generated by mating an *ST3Gal1-floxed* mouse with a mouse expressing Cre driven by a lymphocyte protein tyrosine kinase promoter (Lck-Cre) (Priatel et al. 2000). Both strains of mice were assessed for T cell development stages including CD4 and CD8, double negative cells (DN), immature CD4/CD8 single positive cells (ISP4/ISP8) that do not express T cell receptor (TCR), double positive CD4/CD8 cells (DP), and mature single positive (SP) CD4 and CD8 T cells expressing T cell receptor (Klein et al. 2014). Notably, in the T cell-specific knockout, ST3Gal1 is absent early in T cell development since the Lck promoter is activated in DN thymocytes (Carow et al. 2016). Thus, in both the systemic and T cell-specific knockout strains, ST3Gal1 is absent in all stages of T cell development. In ST3Gal1 deficient strains there was little impact on the early stage thymocyte development, except for an expansion of early immature CD8 single positive cells (ISP8) that are precursors of CD4/CD8 double positive cells (Priatel et al. 2000; Moody et al. 2001; Kao et al. 2006). The most significant difference between the wild type and ST3Gal1 knockout mice was a deficiency in the peripheral mature CD8^+^ T cells resulting from apoptosis, which was not seen with CD4^+^ T cells. Initially, it was suggested that the CD8^+^ T cells exhibited increased non-TCR-dependent (non-cognate) binding to major histocompatibility complex (MHC) class 1 (Moody et al. 2001), but it was later found to be a property of the expanded ISP8 cells that were disproportionately represented in the single positive CD8 cell population. Non-cognate binding in the ST3Gal1-deficient mature CD8^+^ T cells was similar to that of wild type CD8^+^ T cells (Kao et al. 2006). The mechanism by which ST3Gal1-deficient CD8^+^ T cells undergo apoptosis is still unknown (Priatel et al. 2000; Kao et al. 2006; Irons et al. 2024).

Systemic and T cell-specific knockout mice have also been developed for ST6Gal1 (Hennet et al. 1998; Marino et al. 2008; Zhang et al. 2025). The T cell-specific knockout was generated using a CD4-Cre, resulting in an ST6Gal1 deficiency in CD4/CD8 DP precursors and beyond. A significant impairment in thymocyte development was demonstrated in mice with the systemic ST6Gal1 knockout, as evidenced by a marked reduction of thymocyte numbers across all developmental stages (Marino et al. 2008). This was not observed for the T cell-specific knockout, suggesting that the reduced numbers of T cells in the systemic knockout resulted from other cells involved in T cell maturation and survival (Zhang et al. 2025). However, T cells in the CD4-Cre mice were less proliferative in response to antibody stimulation in vitro and exhibited a dampened immune response in MC38 colon tumor-bearing mice (Zhang et al. 2025).

With a goal to evaluate the importance of ST3Gal1 and ST6Gal1 in the activity of mature T cells in cancer immune responses and avoid concerns about the roles of these enzymes in T cell development, we have generated two transgenic mouse lines in which ST3Gal1 (T-ST3KO) or ST6Gal1 (T-ST6KO) is conditionally deleted in mature T cells. This was accomplished by mating floxed-*ST3Gal1* or floxed-*ST6Gal1* mice with distal Lck promoter-mediated Cre (dLck-Cre) mice. Unlike Lck-Cre and CD4-Cre described above, which are activated at the DN and DP stages of development, respectively, the dLck promoter is activated later in the selection of SP CD4 and CD8 T cells (Zhang et al. 2005; Figueroa et al. 2021). We characterize the impact of the deletion of ST3Gal1 and ST6Gal1 on mature CD4 and CD8 T cells in the thymus, spleen, and lymph nodes, and the functional consequences of their absence in immune responses controlling the progression of MC38 solid tumors and lung metastasis of B16F10 melanoma.

## 2. Results

### 2.1. Generation of mature T cell-specific ST3Gal1 or ST6Gal1 knockout mice

Mature T cell-specific ST3Gal1 or ST6Gal1 knockout mouse strains were obtained by crossing floxed-*ST3Gal1* (ST3Gal1^fl/fl^) (Priatel et al. 2000), which were kindly gifted by Dr. Jamey Marth from Sanford Burnham Prebys Medical Discovery Institute, or floxed-*ST6Gal1* (ST6Gal1^fl/fl^) (Collins et al. 2004; Oswald et al. 2020) mice generously gifted by Dr. Brian Cobb at Case Western Reserve University, with dLck-Cre mice, to yield the desired T-ST3KO and T-ST6KO mice, respectively. The exon 2 of the *St3gal1* or *St6gal1* gene was deleted in the homozygous floxed mice carrying dLck-Cre. The homozygous T-ST3KO and T-ST6KO mice were identified by genotyping using specific primers (Supplementary Fig. S1A-D). The T-ST3KO mice differ from those previously described generated using Lck-Cre mice (Priatel et al. 2000) because the Lck promoter is expressed early in T cell development, while the dLck promoter used here is activated only in the late-stage SP thymocytes (Zhang et al. 2005). Importantly, since the T-ST3KO and T-ST6KO mice were generated from different wild type (WT) strains (ST3Gal1^fl/fl^ or ST6Gal1^fl/fl^), in all experiments we use the corresponding parental WT for comparison.

### 2.2. Normal thymic development of T cells in T-ST3KO mice

**Figure 1:**
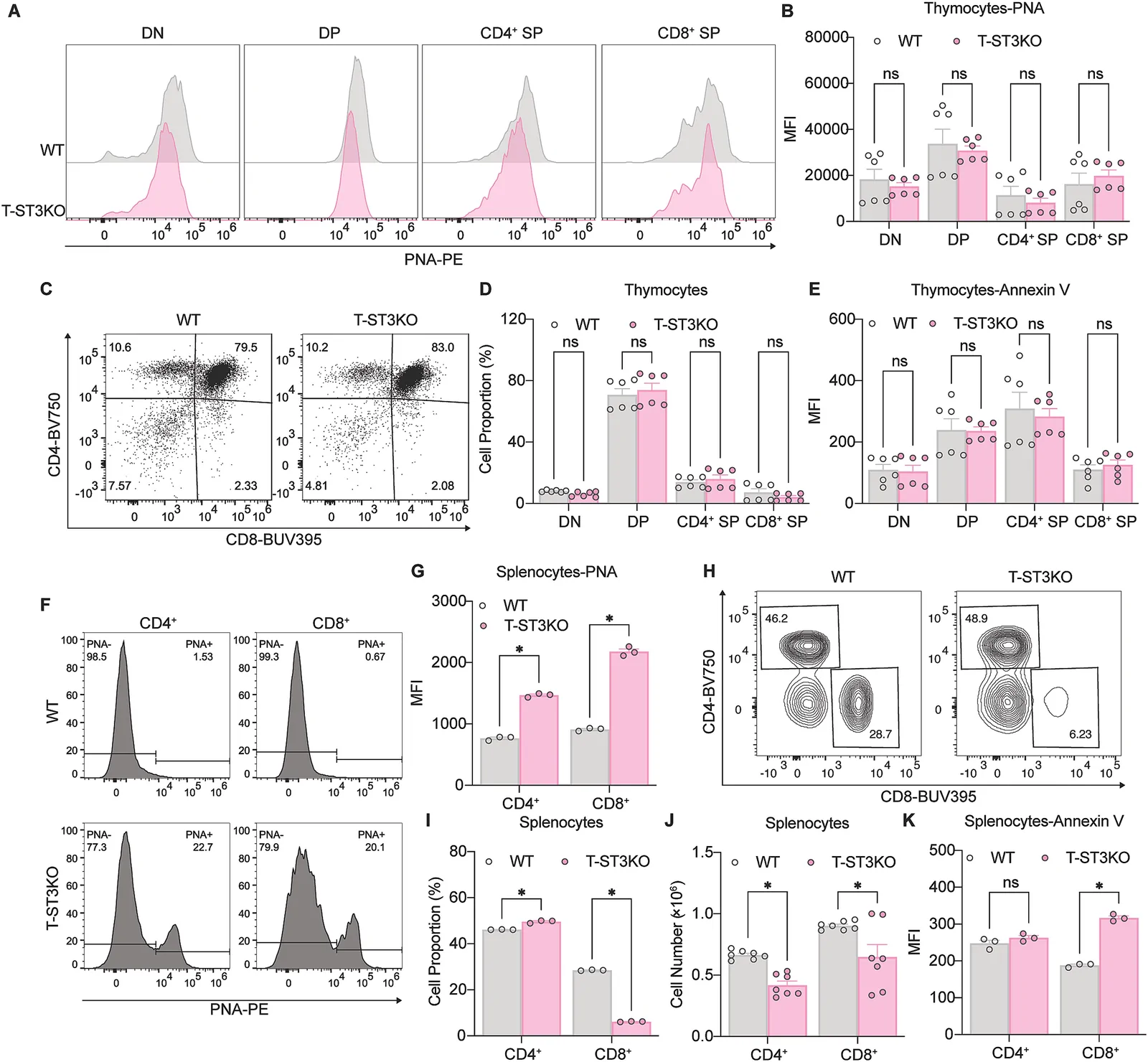
T cells of T-ST3KO mice exhibit normal thymic development and enhanced peripheral apoptosis compared to their parental WT mice. **(A-E)** Analysis of thymic T cells. **(A)** Flow cytometry histograms of thymocytes from WT and dLck-Cre generated T-ST3KO mice stained with PE-labeled PNA (PNA-PE) gated for CD4^−^CD8^−^ double negative (DN), CD4^+^CD8^+^ double positive (DP), CD4^+^ single-positive (SP), and CD8^+^ SP T cells. **(B)** Mean fluorescence intensity (MFI) for PNA-PE in panel A. **(C**) Flow cytometry dot plot analysis of thymocytes of WT and T-ST3KO mice for expression of CD4 and CD8. **(D)** Live T cell proportions. **(E)** MFI of thymocytes stained with annexin V (PE/Cy7-labeled). **(F-K)** Analysis of splenic T cells. **(F)** Flow cytometry histograms of PNA-PE stained splenic CD4^+^ and CD8^+^ T cells. **(G)** MFI for PNA-PE in panel F. **(H**) Flow cytometry contour plot of splenic CD4^+^ and CD8^+^ T cells. **(I)** Relative proportions of cells from panel H. **(J)** Total number of splenic CD4^+^ and CD8^+^ T cells. **(K)** MFI of splenic CD4^+^ and CD8^+^ T cells stained with annexin V (PE/Cy7-labeled). Cells were gated from live populations and data presented as mean ± SEM. Comparisons were analyzed by multiple unpaired t-tests corrected by the Holm-Šidák method. Differences are considered statistically significant at ^*^  *P* ≤ 0.05; ns, not significant (*P* > 0.05).

Since peanut agglutinin (PNA) is used as a marker for the ST3Gal1-mediated conversion of PNA^+^ immature T cells to PNA^−^ mature T cells, we compared the binding of PNA to T cell populations from the thymus of T-ST3KO and parental WT mice (Fig. 1A). Binding of PNA was comparable for WT and T-ST3KO mice at all developmental stages (Fig. 1A and B), including CD4^−^CD8^−^ double-negative (DN), CD4^+^CD8^+^ double-positive (DP), and CD4^+^ or CD8^+^ single-positive (SP) cells. This contrasts with the systemic ST3Gal1 knockout and the T cell-specific Lck-Cre-mediated ST3Gal1 knockout strains reported by Priatel et al. (Priatel et al. 2000), which showed greater PNA^+^ staining in the SP cells. The similarity in the histograms of the WT and T-ST3KO mice is consistent with activation of the dLck promoter late in the positive selection for the CD4^+^ and CD8^+^ SP cells (Figueroa et al. 2021), and the fact that ST3Gal1 already produced will continue to be active for several days when the mature cells emigrate from the thymus to the periphery (Weinreich and Hogquist 2008). The weight of the thymus (Table 1), the total number of thymocytes (Table 2), the proportions and the level of detectable annexin V, a measure of apoptosis, were also equivalent in the T-ST3KO and parental WT mice across all developmental stages (Fig. 1C-E).

**Table 1:** Organ weight (mg).

	WT (ST3Gal1^fl/fl^)	T-ST3KO	WT (ST6Gal1^fl/fl^)	T-ST6KO
Thymus	68.8 ± 20.6	77.1 ± 20.6	64.4 ± 10.7	68.0 ± 28.1
Spleen	74.8 ± 14.0	69.0 ± 15.2	75.1 ± 3.7	68.4 ± 8.4

**Table 2:** Total cell number (×10^6^).

	WT (ST3Gal1^fl/fl^)	T-ST3KO	WT (ST6Gal1^fl/fl^)	T-ST6KO
Thymocyte	10.4 ± 3.7	8.9 ± 3.3	7.0 ± 1.7	7.9 ± 2.0
Splenocyte	7.5 ± 2.7	7.1 ± 1.5	8.9 ± 1.5	9.0 ± 1.3

### 2.3. **Reduced peripheral CD8**  ^**+**^  **T cells in T-ST3KO mice**

In spleen (Fig. 1F-K) and lymph nodes (Supplementary Fig. S2A-F), CD4^+^ and CD8^+^ T cells from WT mice (ST3Gal1^fl/fl^) were 100% PNA^−^ as expected (Gillespie et al. 1993; Priatel et al. 2000). In the T-ST3KO mice, CD4^+^ and CD8^+^ T cells exhibited increased binding of PNA reflecting the loss of ST3Gal1. However, only 20%–40% of the cells in the spleen and lymph nodes were PNA^high^ (Fig. 1F, G; Supplementary Fig. S2A and B). Also striking is the reduced number of both CD4^+^ and CD8^+^ cells relative to WT, with a 25% reduction in CD8^+^ cells in the spleen and > 80% in lymph nodes (Fig. 1J; Supplementary Fig. S2). The reduced number of T cells corresponds to an increase in the expression of annexin V, indicating enhanced apoptosis (Fig. 1K; Supplementary Fig. S2F). Thus, we conclude that the dLck-Cre deletion of the floxed ST3Gal1 gene is not completely penetrant and combined with increased apoptosis of the cells without ST3Gal1, they become the dominant in the periphery. These findings are consistent with previously reported systemic ST3Gal1 knockout and Lck-Cre-mediated ST3Gal1 knockout mice (Priatel et al. 2000). Our results suggest that ST3Gal1 expression in mature T cells is essential for their survival in the periphery and is not a consequence of a defect in their differentiation in the thymus.

### 2.4. **Normal development and distribution of CD4**  ^**+**^  **and CD8**^**+**^  **T cells in T-ST6KO mice**

**Figure 2:**
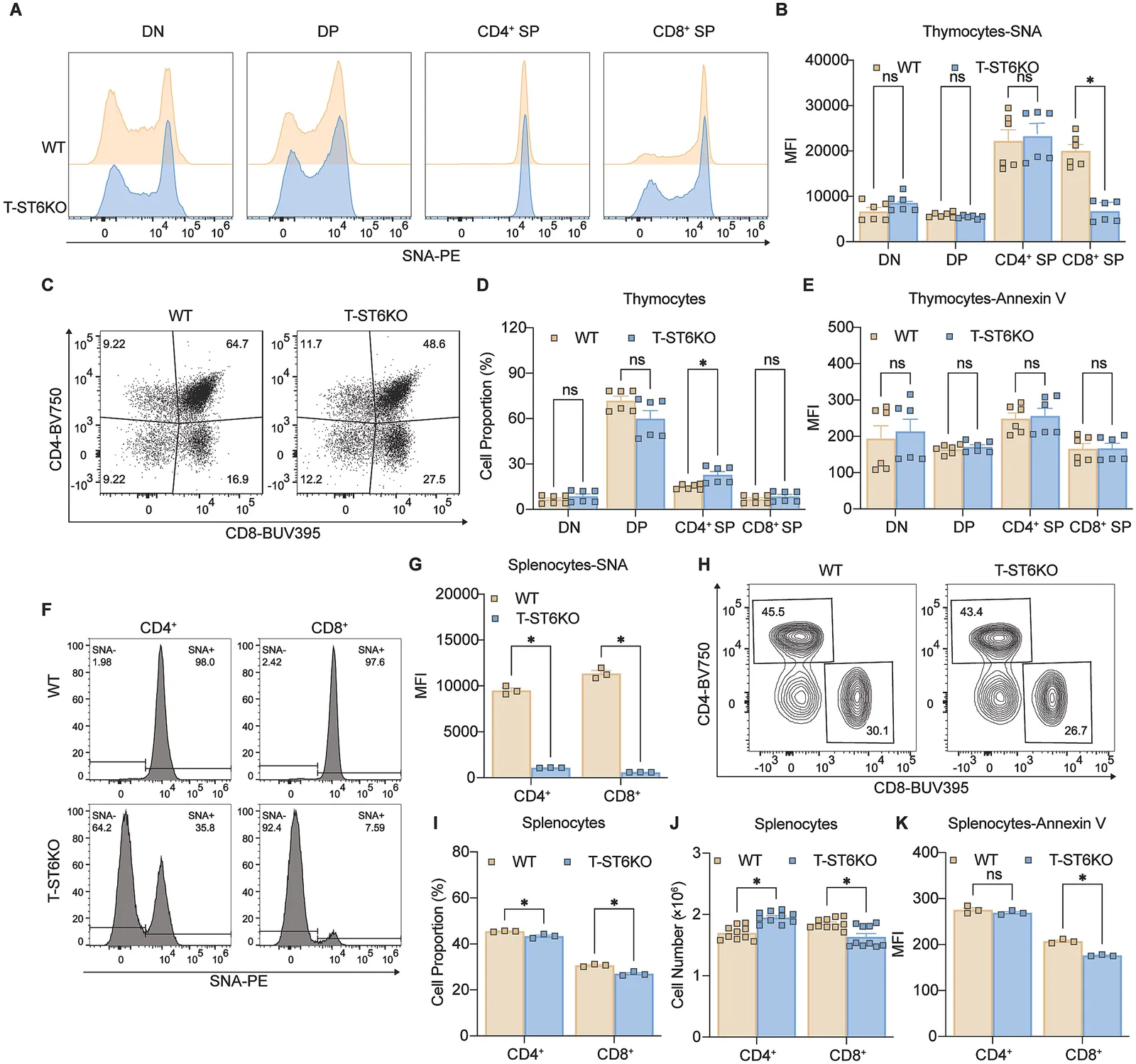
T cells of T-ST6KO mice exhibit minimal differences from parental WT in thymus and spleen. **(A-E)** Analysis of thymic T cells. **(A)** Flow cytometry histograms of thymocytes from WT and dLck-Cre generated T-ST6KO mice stained with PE-labeled SNA (SNA-PE) gated for CD4^−^CD8^−^ double negative (DN), CD4^+^CD8^+^ double positive (DP), CD4^+^ single-positive (SP), and CD8^+^ SP T cells. **(B)** Mean fluorescence intensity (MFI) for SNA-PE in panel A. **(C)** Flow cytometry dot plot analysis of thymocytes of WT and T-ST6KO mice for expression of CD4 and CD8. **(D)** Live T cell proportions. **(E)** MFI of thymocytes stained with annexin V (PE/Cy7-labeled). **(F-K)** Analysis of splenic T cells. **(F)** Flow cytometry histograms of SNA-PE stained splenic CD4^+^ and CD8^+^ T cells. **(G)** MFI for SNA-PE in panel F. **(H**) Flow cytometry contour plot of splenic CD4^+^ and CD8^+^ T cells. **(I)** Relative proportions of cells from panel H. **(J)** Total number of splenic CD4^+^ and CD8^+^ T cells. **(K)** MFI of splenic CD4^+^ and CD8^+^ T cells stained with annexin V (PE/Cy7-labeled). Cells were gated from live populations and data presented as mean ± SEM. Comparisons were analyzed by multiple unpaired t-tests corrected by the Holm-Šidák method. Differences are considered statistically significant at ^*^  *P* ≤ 0.05; ns, not significant (*P* > 0.05).

Since the product of ST6Gal1, Siaα2,6Galβ1–4GlcNAc, is recognized by *S. nigra* agglutinin (SNA), we compared SNA binding to thymic T cells from T-ST6KO and parental WT mice (Fig. 2A and B). Only CD8^+^ SP thymocytes showed a decrease of SNA binding. This is consistent with the activation of dLck-Cre late in the selection of single-positive cells, and that CD8^+^ T cells mature more slowly and take several days longer to emigrate from the thymus (Weinreich and Hogquist 2008; Figueroa et al. 2021; Baldwin and Robey 2024). Moreover, relative to the parental WT mice, the weight of the thymus (Table 1), the total number of thymocytes (Table 2), and the relative percentages, cell numbers, and expression of annexin V, across all developmental stages of thymocytes (Fig. 2C-E) were unaltered, suggesting limited effects of the dLck-Cre mediated deletion of ST6Gal1 in T cell development in the T-ST6KO mice. Previous studies with CD4-Cre mediated ST6Gal1 knockout mice also saw no impact on thymic T cell development despite activation of the CD4-Cre promoter at the earlier DP stage (Zhang et al. 2025). However, in the systemic ST6Gal1 knockout, reduction in SNA was apparent at the earliest stages, and there was a reduced cellularity in the thymus (Hennet et al. 1998; Marino et al. 2008).

Peripheral T cells in spleen and lymph nodes showed a strong reduction in binding of SNA (Fig. 2F and G; Supplementary Fig. S2G and H). Approximately 90% of CD8^+^ T cells and 60% of CD4^+^ T cells were SNA^−^ in both compartments. Relative to the parental WT mice, there was little change in spleen weight (Table 1) and splenocyte numbers (Table 2), and only small fluctuations in cell proportions, numbers and annexin V levels (Fig. 2H-K and Supplementary Fig. S2I-L). Similar results were seen with the MC38 tumor model in the CD4-Cre generated ST6Gal-I deficient mice (Zhang et al. 2025).

### 2.5. **Enhanced in vitro activation of CD8**  ^**+**^  **T cells from T-ST3KO mice**

**Figure 3:**
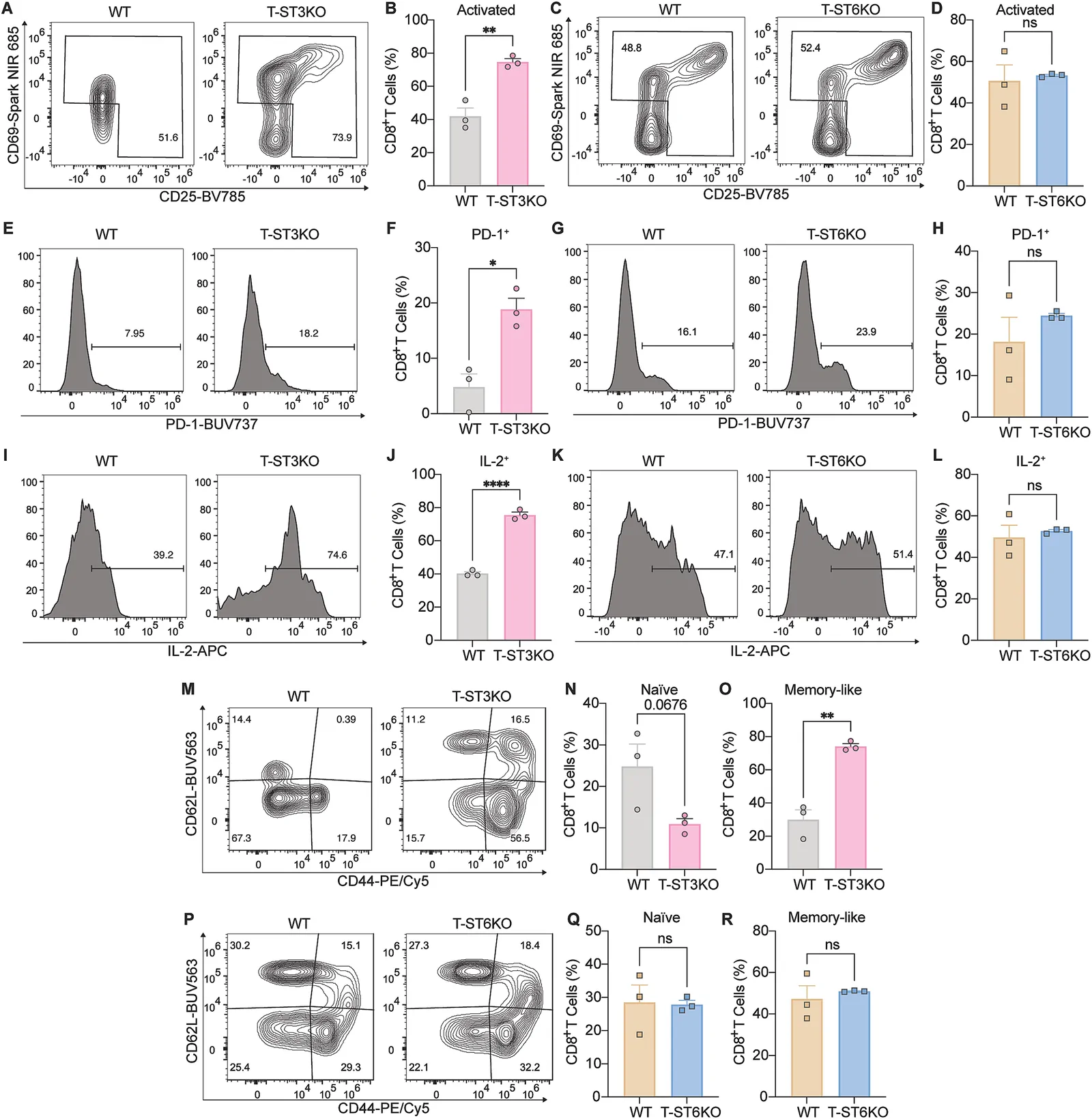
CD8^+^ T cells of T-ST3KO mice, but not T-ST6KO mice, display enhanced in vitro activation. Splenic T cells were isolated and purified from 6–8-week-old T-ST3KO, T-ST6KO, and their parental WT mice, and cultured with soluble anti-CD28 in an anti-CD3 pre-coated flat-bottom 96 well plate at a density of 0.1 M/well for 48 h. T cells from ST3Gal1^fl/fl^ and ST6Gal1^fl/fl^ mice were used as the corresponding WT mice for T-ST3KO and T-ST6KO mice, respectively. **(A)** Flow cytometry contour plot of activated (defined as CD25^high^ and/or CD69^high^) WT/T-ST3KO CD8^+^ T cells. **(B)** Relative proportions of cells from panel A. **(C)** Flow cytometry contour plot of activated WT/T-ST6KO CD8^+^ T cells. **(D)** Relative proportions of cells from panel C. **(E)** Flow cytometry histogram of WT/T-ST3KO CD8^+^ T cells stained with BUV737-labeled anti-PD-1. **(F)** Relative proportions of PD-1 positive cells from panel E. **(G)** Flow cytometry histogram of WT/T-ST6KO CD8^+^ T cells stained with BUV737-labeled anti-PD-1. **(H)** Relative proportions of PD-1 positive cells from panel G. **(I)** Flow cytometry histogram of WT/T-ST3KO CD8^+^ T cells stained with APC-labeled anti-IL-2. **(J)** Relative proportions of IL-2 positive cells from panel I. **(K)** Flow cytometry histogram of WT/T-ST6KO CD8^+^ T cells stained with APC-labeled anti-IL-2. **(L)** Relative proportions of IL-2 positive cells from panel K. **(M)** Flow cytometry contour plot of naïve (defined as CD44 negative and CD62L positive) and memory-like (defined as CD44 positive) WT/T-ST3KO CD8^+^ T cells. **(N** and **O)** Relative proportions of naïve subset (N) and memory-like subset (O) from panel M. **(P)** Flow cytometry contour plot of naïve and memory-like WT/T-ST6KO CD8^+^ T cells. **(Q and R)** Relative proportions of naïve subset (Q) and memory-like subset (R) from panel P. Data are presented as mean ± SEM. Comparisons were analyzed by unpaired two-tailed Student’s t-tests. Differences are considered statistically significant at ^*^  *P* ≤ 0.05, ^**^  *P* ≤ 0.01, and ^****^  *P* ≤ 0.0001; ns, not significant (*P* > 0.05).

To assess the impact of ST3Gal1 or ST6Gal1 deficiency on T cell activation, we stimulated isolated splenic T cells with anti-CD3 and either anti-CD28 (Fig. 3) or CD80-Fc (Supplementary Fig. S3) for 48 h. CD8^+^ T cells were assessed for activation, defined as CD25^high^ and/or CD69^high^. The T cells from T-ST3KO and T-ST6KO mice were compared for their activation with T cells from their corresponding ST3Gal1^fl/fl^ or ST6Gal1^fl/fl^ parental strains. Relative to WT CD8^+^ T cells, T-ST3KO CD8^+^ T cells showed enhanced activation, and T-ST6KO CD8^+^ T cells showed equivalent activation (Fig. 3A-D; Supplementary Fig. S3A-D). Enhanced activation was also only observed for T-ST3KO CD8^+^ T cells for elevated expression of programmed cell death protein 1 (PD-1) and interleukin-2 (IL-2) (Fig. 3E-L; Supplementary Fig. S3E-L). Upon antigen encounter and activation, naïve T cells differentiate into memory cells to maintain their capacity for proliferation, self-renewal, and effector functions (Kaech et al. 2012). In keeping with their enhanced activation, T-ST3KO CD8^+^ T cells displayed a greater reduction in the naïve subset (defined as CD44 negative and CD62L positive) and an increase in memory-like cells (defined as CD44 positive), whereas T-ST6KO CD8^+^ T cells showed naïve and memory subset levels equivalent to those from their parental WT (Fig. 3M-R; Supplementary Fig. S3M-R). CD4^+^ T cells were also assessed for these parameters under anti-CD3 plus anti-CD28 stimulation, where no differences were seen between CD4^+^ T cells from the T-ST3KO or T-ST6KO strains from their corresponding parental WT (Supplementary Fig. S4).

Since previous reports had shown enhanced activation of T cells upon treatment with neuraminidase (Brennan et al. 2006; Nan et al. 2007; Edgar et al. 2021; Yang et al. 2024), we included a positive control with vibrio cholera (VC) sialidase with the parental WT cells in experiments using anti-CD3 and CD80-Fc for activation (Supplementary Fig. S3). Interestingly, VC sialidase enhanced the activation of CD8^+^ T cells from both WT strains (ST3Gal1^fl/fl^ and ST6Gal1^fl/fl^). Activation of the WT + VC cells increased to levels observed in T-ST3KO CD8^+^ T cells, suggesting that the increased activation of the T-ST3KO cells resulted from the sialic acid deficiency. In contrast, WT + VC activation was consistently greater than that of either the WT or T-ST6KO CD8^+^ T cells. We therefore assessed the affinity of CD28 binding to CD80 in WT, T-ST3KO, and T-ST6KO CD8^+^ T cells. Complementing the activated phenotypes, T-ST3KO CD8^+^ T cells exhibited increased CD28-CD80 binding, whereas no difference was observed in T-ST6KO CD8^+^ T cells (Supplementary Fig. S5A and B).

### 2.6. Absence of enhanced control of MC38 tumors in T-ST3KO and T-ST6KO mice

**Figure 4:**
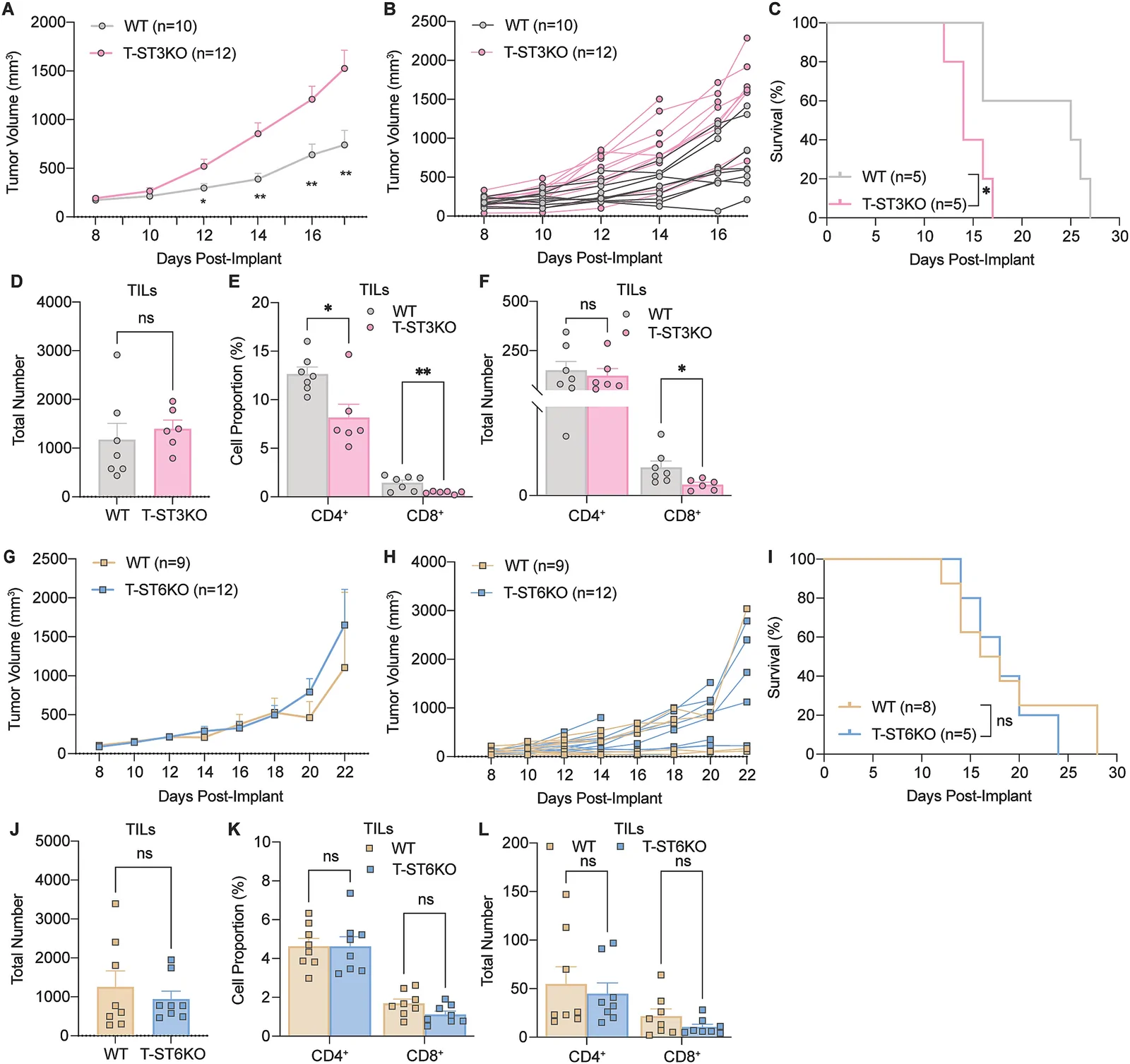
MC38 subcutaneous tumors exhibit more aggressive growth in T-ST3KO mice, but not in T-ST6KO mice. Single-cell suspensions (0.5 million) were injected subcutaneously in the right leg of 6–8-week-old female T-ST3KO **(A-F)** or T-ST6KO **(G-L)** or their parental WT mice on day 0. Tumor size was subsequently monitored, and the number of infiltrated immune cells (TILs) was quantified in each tumor at the endpoint. **(A** and **B)** Average (A) and individual mouse (B) MC38 tumor growth curves in T-ST3KO and WT mice. **(C)** Kaplan–Meier survival analysis. **(D)** Tumor-infiltrating CD45^+^ leukocytes (TILs) total number. **(E** and **F)** The proportions (E) and the total number (F) of tumor-infiltrating CD4^+^ T cells and CD8^+^ T cells. **(G** and **H)** Average (G) and individual mouse (H) MC38 tumor growth curves in T-ST6KO and WT mice. **(I)** Kaplan–Meier survival analysis. **(J)** TILs total number. **(K** and **L)** The proportions (K) and the total number (L) of tumor-infiltrating CD4^+^ T cells and CD8^+^ T cells. Data are presented as mean ± SEM. The survival curves were analyzed using the Kaplan–Meier method and compared statistically using the log-rank (mantel-cox) test. Comparisons were analyzed by unpaired two-tailed Student’s t-tests for panels D and J, while by multiple unpaired t-tests corrected by the Holm-Šidák method for other panels. Differences are considered statistically significant at ^*^  *P* ≤ 0.05, and ^**^  *P* ≤ 0.01; ns, not significant (*P* > 0.05).

The MC38 subcutaneous tumor model is an immunologically hot murine colon adenocarcinoma known for recruiting inflammatory T cells (Schetters et al. 2020). Growth of MC38 tumors was more aggressive in T-ST3KO mice compared to the parental WT mice (Fig. 4A-F). Subcutaneous implantation of MC38 cells resulted in rapid formation of solid tumors, rapid growth, and poor survival in T-ST3KO mice compared to parental WT mice (Fig. 4A-C). Although there was strong recruitment of tumor-infiltrating CD45^+^ leukocytes (TILs), the proportion of CD8^+^ T cells was significantly reduced (Fig. 4D-F). The reduced number of CD8^+^ T cells in the tumor is commensurate with that in the spleen and lymph nodes (Fig. 1J; Supplementary Fig. S2E). Therefore, the more aggressive tumor growth could simply reflect reduced T cell control compared to that of the parental WT mouse.

By contrast, with the MC38 model there were no significant differences between T-ST6KO and parental WT mice in tumor growth, survival, or tumor-infiltrating T cell populations (Fig. 4G-L). In this case, despite the loss of ST6Gal1, there are similar numbers of CD8^+^ T cells, and tumor control is equivalent to that of the parental WT mice, suggesting that the T-ST6KO T cells are equally active in tumor suppression. In this regard, Zhang et al. similarly found that MC38 tumors grew equivalently in the parental WT and CD4-Cre generated ST6Gal1 knockout (Zhang et al. 2025).

We also evaluated the B16F10 melanoma as a subcutaneous solid tumor, a model considered immunologically cold (Leick et al. 2019). There was no difference in tumor progression in WT, T-ST3KO, and T-ST6KO mice bearing B16F10 or in the level of TILs and T cells (Supplementary Fig. S6A-L).

### 2.7. B16F10 pulmonary metastasis is more aggressive in T-ST6KO mice

**Figure 5:**
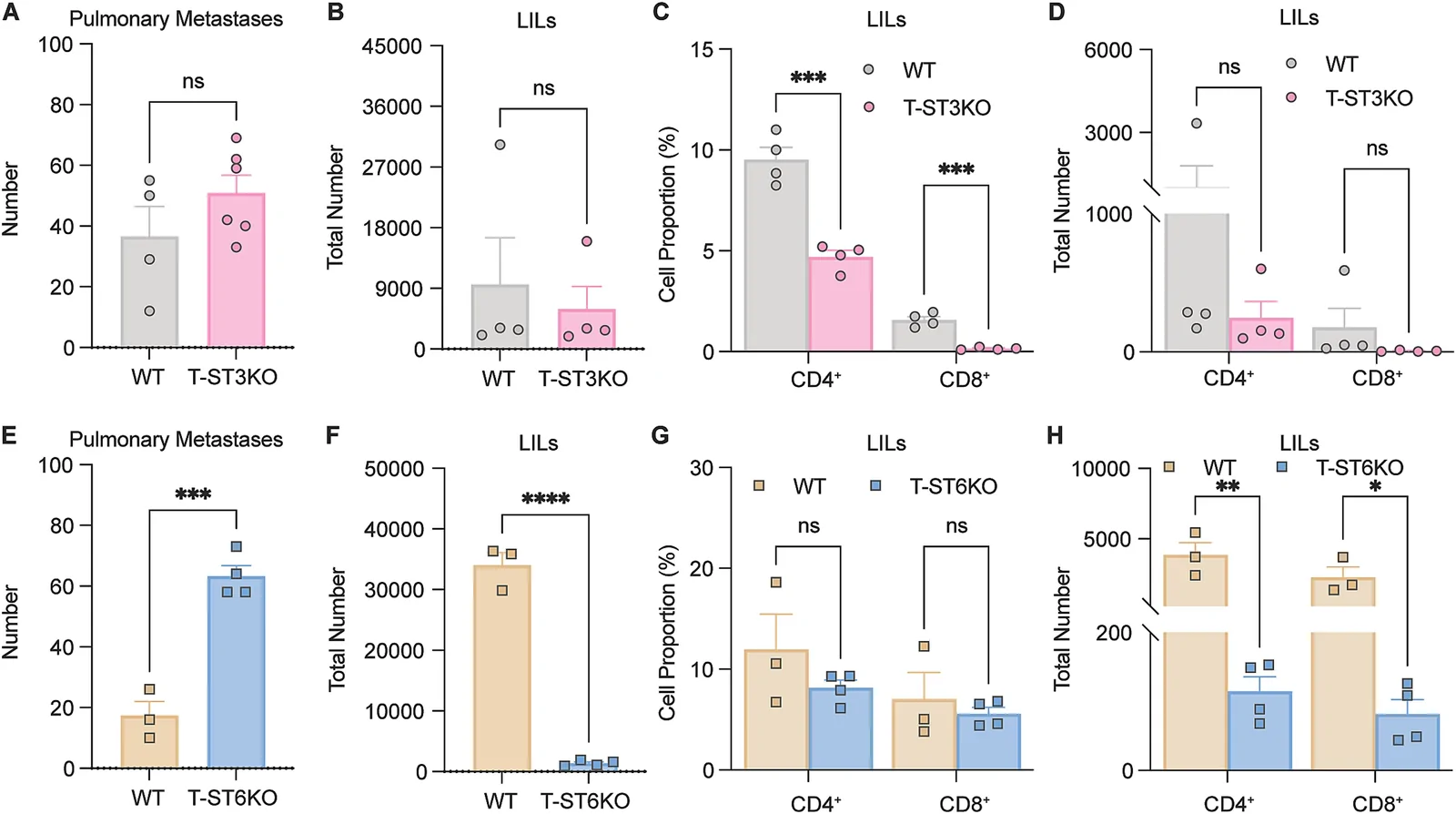
B16F10 tumor cells display more aggressive pulmonary metastasis in T-ST6KO mice, but not in T-ST3KO mice. Single-cell suspensions of B16F10 cells (1x10^5^) were tail vein injected in 6–8-week-old T-ST3KO **(A-D)** or T-ST6KO **(E-H)** males or their parental WT mice on day 0. Lungs were harvested on day 18 for further analysis, and the infiltrated immune cell numbers in each lung were quantified. **(A)** Quantification of pulmonary metastatic foci. **(B)** Lung-infiltrating CD45^+^ leukocytes (LILs) total number. **(C** and **D)** The proportions (C) and the total number (D) of lung-infiltrating CD4^+^ T cells and CD8^+^ T cells. **(E)** Quantification of pulmonary metastatic foci. **(F)** LILs total number. **(G** and **H)** The proportions (G) and the total number (H) of lung-infiltrating CD4^+^ T cells and CD8^+^ T cells. Data are presented as mean ± SEM. Comparisons were analyzed by unpaired two-tailed Student’s t-tests for panels a, B, E and F, while by multiple unpaired t-tests corrected by the Holm-Šidák method for other panels. Differences are considered statistically significant at ^*^  *P* ≤ 0.05, ^**^  *P* ≤ 0.01, ^***^  *P* ≤ 0.001, and ^****^  *P* ≤ 0.0001; ns, not significant (*P* > 0.05).

The B16F10 melanoma is administered intravenously for evaluation in a pulmonary metastasis model, where T cell infiltration dynamics play a different role. In this model, we saw no difference in the number of metastases between the parental WT and T-ST3KO mice (Fig. 5A) and the total numbers of CD45^+^, CD4^+^, and CD8^+^ T cells in the lung tissue were also comparable (Fig. 5B-D). In contrast, T-ST6KO mice exhibited increased pulmonary metastatic nodules compared to the parental WT mice (Fig. 5E), which was accompanied by reduced infiltration of CD45^+^ leukocytes, CD4^+^, and CD8^+^ T cells in lung tissue (Fig. 5F-H).

## 3. Discussion

Previous reports have described the impact of the genetic deficiency of ST3Gal1 and ST6Gal1 on T cell immune responses in knockout mice where the genes were deleted systemically, or conditionally in T cells at early stages of development (Hennet et al. 1998; Priatel et al. 2000; Marino et al. 2008; Zhang et al. 2025). Here we report the development of mouse strains conditionally deficient in the ST3Gal1 and ST6Gal1 genes using parental mice with the floxed genes (flanked by *LoxP* sites) mated with dLck-Cre mice expressing the Cre recombinase late in T cell development, specifically in the late stage of selection of mature (SP) CD4^+^ and CD8^+^ T cells (Zhang et al. 2005; Baldwin and Robey 2024). The motivation was to determine what phenotypes of these deficiencies were independent of the developmental roles of these two enzymes. Consistent with the late expression of the dLck promoter, T cell development in the thymus of both the T-ST3KO and T-ST6KO mice was normal, with only minimal changes in the loss of their sialylated products, as detected with PNA and SNA, respectively (Fig. 1 and 2). While all mouse strains were on a C57BL/6 background, their respective wild type parental strains (WT) with the ST3Gal1^fl/fl^ or ST6Gal1^fl/fl^ genes were separately maintained. Accordingly, we used these respective WT strains in experiments for comparison with the impact of sialyltransferase deletion on T cell development and function in the T-ST3KO and T-ST6KO mice.

One major phenotype of the ST3Gal1 knockout mice seen previously with the systemic and conditional Lck-Cre knockout mice was the reduced numbers of CD8^+^ T cells in the periphery, coinciding with the increase in the expression of annexin V, a measure of apoptosis (Priatel et al. 2000). The reduced number of CD8^+^ T cells is also seen in the T-ST3KO, with a reduction of 30%–84% of CD8^+^ T cells in spleen and lymph nodes despite the incomplete penetrance of the ST3Gal1 knockout as evidenced by the substantial numbers of PNA^−^, which are effectively WT cells. Importantly, in systemic ST3Gal1 knockout (Priatel et al. 2000), and in a Lck-Cre derived sialic acid monophosphate cytidine synthetase (Cmas) knockout that eliminates the synthesis of all sialosides (Schmidt et al. 2024), penetrance was largely complete, and while there were normal levels of CD8^+^ T cells in the thymus, there was >90% reduction in the periphery. Taken together, these results strongly suggest that apoptosis of CD8^+^ T cells occurs following emigration from the thymus.

In contrast, CD8^+^ T cells in T-ST6KO were present at near-normal numbers in the periphery (spleen and lymph nodes), despite near-complete penetrance of the deficiency, as evidenced by the lack of SNA binding (Fig. 2, Supplementary Fig. S2). Why T cell survival in the periphery is so dramatically affected by the ST3Gal1 knockout but not by the ST6Gal1 knockout remains unclear. However, it is notable that mucin glycoproteins such as CD45 and CD43 are the most abundant glycoproteins on the surface of the T cell, and that ST3Gal1 is considered to be responsible for the majority of sialic acids on O-glycans on these proteins (Clark and Baum 2012; Irons et al. 2024). Since ST6Gal1 is primarily responsible for sialylation of N-glycans, there will be minimal impact on mucin-type glycoproteins. Thus, CD8^+^ T cells in T-ST3KO and T-ST6KO mice will differ in the amount of sialic acid lost which surface glycoproteins are impacted.

Relative to T cells from their corresponding WT, T-ST3KO and T-ST6KO T cells also differed in their activation in vitro (Fig. 3, Supplementary Fig. S3). CD8^+^ T cells from T-ST3KO mice exhibited enhanced activation, despite the reduced number and enhanced apoptosis of peripheral CD8^+^ T cells, and the fact that the penetrance of the ST3Gal1 knockout was incomplete. By contrast, the in vitro activation of ST6Gal1-deficient T cells was equivalent to that of their parental WT T cells. In this regard, similar results were observed for the activation of T cells from CD4-Cre-driven ST6Gal1 knockout mice (Zhang et al. 2025), using in vitro activation conditions similar to those used here. However, under weaker activation conditions, ST6Gal1-deficient T cells showed significantly reduced activation relative to WT T cells. Thus, taken as a whole, compared to the parental WT T cells, activation of T cells from ST3Gal1 knockout is clearly enhanced, while activation of T cells from ST6Gal1 knockout is equivalent or slightly reduced.

Despite enhanced T cell activation in T-ST3KO mice, the mice were notably deficient in controlling inflammatory MC38 tumors compared to WT mice, resulting in much more aggressive tumor growth and reduced survival (Fig. 4). We attribute this to the reduced numbers of peripheral CD8^+^ T cells with corresponding reduced infiltration in the tumor. Thus, the enhanced in vitro activation did not compensate for the reduced tumor control. We found no difference in the growth of MC38 tumors between T-ST6KO mice and their corresponding WT controls. This result is consistent with similar numbers of CD8^+^ T cells in the WT and T-ST6KO mice, their comparable activation in vitro, and their recruitment into the tumor (TILs). Importantly, however, in the CD4-Cre mediated T cell-specific ST6Gal1 knockout mice, Zhang et al. found that subcutaneous MC38 tumors grew more aggressively than the WT mice (Zhang et al. 2025). In their model, the tumors were growing substantially slower than in ours, so the small reduction in activation of the ST6Gal1 knockout T cells they observed could account for this difference in T cell-mediated control of the tumor compared to the WT mice.

Interestingly, in the immunologically “silent” B16F10 melanoma subcutaneous tumor model (Mosely et al. 2017), there was no significant difference in growth in the T-ST3KO and T-ST6KO compared to the corresponding WT. However, in the melanoma metastasis model with the same cell line, while there was no difference in the number of metastases between the WT and T-ST3KO mice, there was a striking increase in metastasis in the T-ST6KO mice compared to the WT control. Moreover, there was a reduction of lung-infiltrating T cells in the T-ST6KO mice. The results suggest a deficiency in the migration of the T-ST6KO T cells into the lung.

In summary, we find that deficiencies in the ST3Gal1 and ST6Gal1 sialyltransferases in mature T cells have a distinct impact on T cell homeostasis, activation, and antitumor immunity. As reported for a systemic or early development T cell ST3Gal1 knockout strains (Priatel et al. 2000), ST3Gal1-dependent α2–3 sialylation in mature T cells appears to be critical for maintaining peripheral CD8^+^ T cell survival. We further find that its absence enhances CD8^+^ T cell activation thresholds. In contrast, ST6Gal1-dependent sialylation is not required for peripheral homeostasis and does not enhance T cell activation. The notable lack of enhanced control of subcutaneous or metastatic tumor models in the T-ST3KO or T-ST6KO mice suggests that targeting these enzymes in T cells only would be of limited value.

Finally, the mechanisms that underlie these T cell phenotypes remain to be elucidated. One important consideration is that deletion of ST3Gal1 or ST6Gal1 will differentially result in the loss of ligands of Siglecs that recognize α2–3 or α2–6 sialosides and increase ligands of galectins that recognize sialo-glycans terminating in galactose. Since these glycan binding proteins are widely expressed in the tumor microenvironment, spleen, thymus and lymph nodes, the impact on T cell functions could be impacted in numerous contexts (Toscano et al. 2007; Pinho et al. 2025). One limitation of our study is that the Th1, Th2 and Th17 subsets were not separately evaluated in the T-ST3KO, T-ST6KO transgenics and their parental WT strains. This is particularly relevant in light of prior work demonstrating that galectins differentially regulate the activation, survival, and polarization of T cell subsets through their interactions with cell surface ligands (Amano et al. 2003; Chen et al. 2009; Su et al. 2011; Cedeno-Laurent and Dimitroff 2012; Sampson et al. 2016; Luo et al. 2018). In particular, galectin-1 has been characterized as an immunoregulatory galectin that binds to polarized T cell subsets as a consequence of their differential cell surface sialylation, and induces apoptosis (Nguyen et al. 2001; Amano et al. 2003; Hernandez et al. 2006; Toscano et al. 2007; Motran et al. 2008; Cedeno-Laurent et al. 2012; Cedeno-Laurent and Dimitroff 2012). Increased ligands on pro-inflammatory TH1- and TH17-differentiated T cells result in greater apoptosis by galectin-1, while TH2 cells were protected because of increased sialylation by ST6Gal1 (Toscano et al. 2007; Cedeno-Laurent and Dimitroff 2012). Future studies combining comprehensive T cell subset profiling with glycomic analyses will therefore be important for understanding how ST3Gal1- and ST6Gal1-dependent glycosylation shapes subset-specific responses and galectin-mediated regulation.

## 4. Materials and methods

### 4.1. Mice

All mice used in our study were 6–10 weeks old C57BL/6J background mice and were housed in the vivarium at The Scripps Research Institute (La Jolla, CA). Experimental procedures adhered to protocols approved by the Institutional Animal Care and Use Committee of The Scripps Research Institute.

ST3Gal1^fl/fl^ mice were kindly gifted by Dr. Peter Aziz and Dr. Jamey Marth from Sanford Burnham Prebys Medical Discovery Institute (Jackson Laboratory deposited #006897). Jackson Laboratory-available ST6Gal1^fl/fl^ mice were gifted by Dr. Brian Cobb at Case Western Reserve University (Jackson Laboratory deposited #006901) (Hennet et al. 1998). The dLck-Cre mice (#012837) were purchased from Jackson Laboratory. This expresses Cre efficiently in CD4^+^ and CD8^+^ T cells, and weakly in innate T cell subsets (γδ T cells, mucosal-associated invariant T cells, and invariant natural killer T cells) (Zhang et al. 2005; Leick et al. 2019; Figueroa et al. 2021). Mature T cell-specific knockout ST3Gal1 mice (termed T-ST3KO) and ST6Gal1 mice (termed T-ST6KO) were generated by crossing the dLck-Cre mice with ST3Gal1^fl/fl^ or ST6Gal1^fl/fl^ mice, respectively. ST3Gal1^fl/fl^ or ST6Gal1^fl/fl^ mice served as control groups are each called WT when used for comparison with their respective knockout strains. Genotyping was performed after 40 cycles of polymerase chain reaction (PCR) using primers from Jackson Laboratory, with denaturation at 95 °C for 30 s, annealing at 58 °C for 30 s, and elongation at 72 °C for 30 s in each cycle.

### 4.2. Cell preparation from the thymus, spleen, and lymph nodes

Thymuses, spleens, or inguinal lymph nodes were harvested, weighed, and processed by crushing using plungers in phosphate-buffered saline (PBS) and washing through 100 μm strainers. The cell pellet was collected and washed with PBS once by centrifugation at 500 × g for 5 min at 4 °C.

For splenocyte preparation, the cell pellets were lysed using 1× red blood cell lysis buffer (diluted in ddH_2_O, Cat#420301, Biolegend) at room temperature for 2 min, then washed with PBS by centrifugation at 500 × g for 5 min at 4 °C. Thymocytes or lysed splenocytes were resuspended in PBS or T cell culture medium for further experiments or further purification (see below). The numbers of thymocytes and splenocytes were counted using the Moxi GO II cell counter (MXG102, ORFLO) at a 1:80 dilution, and the total cell numbers were calculated.

### 4.3. Cell surface protein and lectin staining

Thymocytes, splenocytes, lymphocytes, isolated splenic T cells, tumor-infiltrating leukocytes (TILs), and lung-infiltrating leukocytes (LILs) were stained with Zombie NIR™ Fixable Viability Kit (1:1000 dilution in PBS, Cat#423106, BioLegend) for 15 min at room temperature in the dark, then blocked with TruStain FcX™ (anti-mouse CD16/32) antibody (1:100 dilution, Cat#423106, BioLegend) for 10 min on ice in flow cytometry staining buffer (FACS buffer: PBS containing 1% BSA and 0.5 mM EDTA). Following blocking, cells were stained with a cocktail of primary antibodies at a 1:600 dilution in FACS buffer for 20 min on ice. BV605-conjugated CD45 (Cat#157217, BioLegend), BV750-conjugated CD4 (Cat#100467, BioLegend), and BUV395-conjugated CD8 (Cat#563786, BD) were used to characterize thymocytes, splenocytes, and lymphocytes from WT, T-ST3KO, and T-ST6KO mice. CD45, CD3, CD4, CD8, described previously, and additional BV785-conjugated CD25 (Cat#423106, BioLegend), Spark NIR 685-conjugated CD69 (Cat#423106, BioLegend), BUV737-conjugated PD-1 (Cat#612791, BD), APC-conjugated IL-2 (Cat#503809, BioLegend), PE/Cy5-conjugated CD44 (Cat#423106, BioLegend), BUV563-conjugated CD62L (Cat#423106, BD) were used to detect splenocytes activation and differentiation in vitro. After twice washing with 100 μl FACS buffer, cells were resuspended in 100 μl FACS buffer, then analyzed by the Cytek® Aurora system (Cytek) and then analyzed using FlowJo™ software.

For lectin staining, after primary antibody staining, cells were stained with lectins (1:100 dilution) pre-complexed with streptavidin-PE (1:1000 dilution) in calcium- and magnesium-containing Dulbecco’s phosphate-buffered saline (DPBS, Cat # 14040117, Gibco) for 20 min on ice in the dark. After lectin staining, cells were washed with FACS buffer and resuspended in 100 μl FACS buffer for subsequent analysis.

### 4.4. T cell isolation, culturing and in vitro T cell activation

T cells were isolated from the splenocytes using EasySep™ Mouse T Cell Isolation Kit (Cat#19851, Stemcell) following the manufacturer’s instructions. T cells were then cultured in the RPMI-1640 medium supplemented with 10% fetal bovine serum (FBS), 1% penicillin and streptomycin (Cat#15140122, Gibco), 50 μM β-mercaptoethanol (Cat#M3148, Sigma), 2 mM L-glutamine (Cat#25030081, Gibco), 1 mM sodium pyruvate (Cat#11360070, Gibco), and 1× MEM non-essential amino acids solution (Cat#11140050, Gibco).

For T cell activation, flat-bottom 96-well plates were coated with 500 ng/mL purified anti-mouse CD3 antibody (clone 17A2, Cat#100201, Biolegend) in 100 μL PBS per well at 37 °C for two hours. Isolated T cells were harvested (by centrifuge) and plated at 0.1 M/well in the pre-coated flat-bottom 96-well plates, along with 5 μg/mL purified anti-mouse CD28 antibody (clone 17.51, Cat#102101, Biolegend) (Fig. 3) or with 250 μg/mL recombinant mouse B7.1 (CD80)-Fc Chimera (carrier-free) (Cat#555406, Biolegend) (Supplementary Fig. S3) at 37 °C in an atmosphere of 5% CO_2_ for 48 h. For vibrio cholerae (VC) sialidase treatment, 5 M/mL isolated T cells were treated at 37 °C for 30 min prior to the activation.

### 4.5. CD80-Fc binding to T cells

Isolated T cells are plated in a round-bottom 96-well plate at a density of 0.1 million/well. After staining with Zombie NIR™ dye to assess viability, cells were blocked with TruStain FcX™ and then incubated with primary antibodies for cell surface markers. Then, stained cells were incubated with recombinant mouse B7.1 (CD80)-Fc Chimera (carrier-free) (Cat#555406, BioLegend) for 20 min on ice for CD80-Fc binding and with R-Phycoerythrin AffiniPure Goat Anti-Human IgG (1:1000 dilution, Cat#109115098, Jackson ImmunoResearch Inc.) for the secondary antibody staining. Cells were washed with FACS buffer between each staining step. All staining steps were performed in the dark.

### 4.6. MC38 and B16F10 tumor models

For the subcutaneous tumor models, a total number of 5 × 10^5^ MC38 cells (in Fig. 4) or B16F10 cells (in Supplementary Fig. S6) were subcutaneously injected into the right flank of 6–8-week-old female or male mice, respectively. Tumors are monitored every two days, and tumor volume was calculated by the formula: length×width×width/2. Tumors were considered to have reached the endpoint when the size exceeded 2000 mm^3^, the mice exhibited abnormal behavior for more than 24 h, or the tumor became ulcerated. Mice reaching the endpoint were recorded for survival analysis and euthanized by CO_2_ overdose followed by cervical dislocation.

For the lung metastasis mouse model in Fig. 5, 1×10^5^ B16F10 cells were injected into the tail vein of male mice on day 0. Lungs were harvested on day 18 for subsequent analysis. Metastatic nodules visible on the surface of the lung tissue were counted and used as the measure of the degree of metastasis.

### 4.7. Tumor and metastatic lungs processing for analysis of tumor-infiltrating leukocytes (TILs) and lung-infiltrating leukocytes (LILs)

Solid tumors or lungs were harvested at the endpoint and preserved in PBS on ice. Tissue was sectioned using sterile scissors, forced through 100 μm strainers, and flushed with PBS or RPMI medium supplemented with 10% FBS. The pellet was lysed using 1× red blood cell lysis buffer (diluted in dH_2_O, Cat#420301, Biolegend) at room temperature for 2 min and washed twice with PBS by centrifugation at 500 × g for 5 min at 4 °C. Cells were resuspended in pre-balanced 5 ml 35% Percoll media (Cat#17089102, Cytiva) and filtered with a 40 μm cell strainer. The cell suspension was gently layered over 67% (bottom) and 44% (middle) Percoll media. The third layer of cells in the Percoll gradient solution was collected after centrifuging at 1000 × g at 20 °C for 25 min. After washing with RPMI-1640 medium containing 10% FBS, the cells were plated in the round-bottom 96-well plates for the subsequent staining. The total numbers of TILs and LILs were counted as Zombie NIR negative and CD45 positive cell groups.

### 4.8. Gating strategies for flow cytometry analysis

Debris and doublets were excluded by gating on a single cell population using forward scatter-area vs. forward scatter-height scatter plots, and the live cells were identified using Zombie NIR™ negative staining. CD4^+^ and CD8^+^ single-positive (SP) T cell subsets, CD4^+^CD8^+^ double-positive (DP) T cell subset, and CD4^−^CD8^−^ double-negative (DN) T cell subsets were gated out from Zombie NIR negative alive cells directly for thymocytes. For splenocytes, lymphocytes, tumor-infiltrating lymphocytes, and lung-infiltrating lymphocytes, all cell subsets were gated from Zombie NIR negative and CD45^+^ cells. Activated T cells (defined as CD25 and/or CD69 positive), programmed cell death protein 1 (PD-1) positive T cells, interleukin-2 (IL-2) positive T cells, naïve T cells (defined as CD44 negative CD62L positive), and memory-like T cells (defined as CD44 positive) were gated from CD8^+^ T cells.

### 4.9. Statistical analysis

All data are presented as mean ± Standard Error of Mean (SEM). Comparisons between two groups were analyzed using unpaired two-tailed Student’s t-tests or multiple unpaired t-tests corrected by the Holm-Šidák method, while comparisons of more than two groups were analyzed by one-way analysis of variance (ANOVA) followed by Fisher’s LSD test. The survival curves were analyzed using the Kaplan–Meier method and compared statistically using the Log-rank (Mantel-Cox) test. Differences are considered statistically significant at ^*^  *P* ≤ 0.05, ^**^  *P* ≤ 0.01, ^***^  *P* ≤ 0.001, and ^****^  *P* ≤ 0.0001; ns, not significant (*P* > 0.05).

## Supplementary Material

Supplementary_materials_cwag065

## Data Availability

All the data is presented as part of the manuscript. Raw data is available upon request.

## References

[ref1] Amado M, Yan Q, Comelli EM, Collins BE, Paulson JC. Peanut agglutinin high phenotype of activated CD8+ T cells results from de novo synthesis of CD45 glycans. J Biol Chem. 2004:279(35):36689–36697. 10.1074/jbc.M405629200. 15210702

[ref2] Amano M, Galvan M, He J, Baum LG. The ST6Gal I sialyltransferase selectively modifies N-glycans on CD45 to negatively regulate galectin-1-induced CD45 clustering, phosphatase modulation, and T cell death. J Biol Chem. 2003:278(9):7469–7475. 10.1074/jbc.M209595200. 12499376

[ref3] Baldwin I, Robey EA. Adjusting to self in the thymus: CD4 versus CD8 lineage commitment and regulatory T cell development. J Exp Med. 2024:221(10):e20230896(1-15). 10.1084/jem.20230896. PMC11232887 38980291

[ref4] Bashian EE, Paulson JC, Wu P. Sialic acids modulate immune responses in cancer: therapeutic opportunities. J Biol Chem. 2026:302(4):111245. 10.1016/j.jbc.2026.111245. 41651427 PMC13010965

[ref5] Baum LG, Derbin K, Perillo NL, Wu T, Pang M, Uittenbogaart C. Characterization of terminal sialic acid linkages on human thymocytes. Correlation between lectin-binding phenotype and sialyltransferase expression. J Biol Chem. 1996:271(18):10793–10799. 10.1074/jbc.271.18.10793. 8631891

[ref6] Boligan KF, Mesa C, Fernandez LE, von Gunten S. Cancer intelligence acquired (CIA): tumor glycosylation and sialylation codes dismantling antitumor defense. Cell Mol Life Sci. 2015:72(7):1231–1248. 10.1007/s00018-014-1799-5. 25487607 PMC11113383

[ref7] Brennan PJ, Saouaf SJ, Van Dyken S, Marth JD, Li B, Bhandoola A, Greene MI. Sialylation regulates peripheral tolerance in CD4+ T cells. Int Immunol. 2006:18(5):627–635. 10.1093/intimm/dxh344. 16291658

[ref8] Bull C, Boltje TJ, Balneger N, Weischer SM, Wassink M, van Gemst JJ, Bloemendal VR, Boon L, van der Vlag J, Heise T, et al. Sialic acid blockade suppresses tumor growth by enhancing T-cell-mediated tumor immunity. Cancer Res. 2018:78(13):3574–3588. 10.1158/0008-5472.CAN-17-3376. 29703719

[ref9] Carow B, Gao Y, Coquet J, Reilly M, Rottenberg ME. Lck-driven Cre expression alters T cell development in the thymus and the frequencies and functions of peripheral T cell subsets. J Immunol. 2016:197(6):2261–2268. 10.4049/jimmunol.1600827. 27503210

[ref10] Cedeno-Laurent F, Dimitroff CJ. Galectin-1 research in T cell immunity: past, present and future. Clin Immunol. 2012:142(2):107–116. 10.1016/j.clim.2011.09.011. 22019770 PMC3266984

[ref11] Cedeno-Laurent F, Watanabe R, Teague JE, Kupper TS, Clark RA, Dimitroff CJ. Galectin-1 inhibits the viability, proliferation, and Th1 cytokine production of nonmalignant T cells in patients with leukemic cutaneous T-cell lymphoma. Blood. 2012:119(15):3534–3538. 10.1182/blood-2011-12-396457. 22383798 PMC3325040

[ref12] Chen HY, Fermin A, Vardhana S, Weng IC, Lo KF, Chang EY, Maverakis E, Yang RY, Hsu DK, Dustin ML, et al. Galectin-3 negatively regulates TCR-mediated CD4+ T-cell activation at the immunological synapse. Proc Natl Acad Sci U S A. 2009:106(34):14496–14501. 10.1073/pnas.0903497106. 19706535 PMC2732795

[ref13] Clark MC, Baum LG. T cells modulate glycans on CD43 and CD45 during development and activation, signal regulation, and survival. Ann N Y Acad Sci. 2012:1253(1):58–67. 10.1111/j.1749-6632.2011.06304.x. 22288421 PMC4190024

[ref14] Coccimiglio M, Chiodo F, van Kooyk Y. The sialic acid-Siglec immune checkpoint: an opportunity to enhance immune responses and therapy effectiveness in melanoma. Br J Dermatol. 2024:190(5):627–635. 10.1093/bjd/ljad517. 38197441

[ref15] Collins BE, Blixt O, DeSieno AR, Bovin N, Marth JD, Paulson JC. Masking of CD22 by cis ligands does not prevent redistribution of CD22 to sites of cell contact. Proc Natl Acad Sci U S A. 2004:101(16):6104–6109. 10.1073/pnas.0400851101. 15079087 PMC395930

[ref16] Comelli EM, Sutton-Smith M, Yan Q, Amado M, Panico M, Gilmartin T, Whisenant T, Lanigan CM, Head SR, Goldberg D, et al. Activation of murine CD4+ and CD8+ T lymphocytes leads to dramatic remodeling of N-linked glycans. J Immunol. 2006:177(4):2431–2440. 10.4049/jimmunol.177.4.2431. 16888005

[ref17] Cornelissen LAM, Blanas A, van der Horst JC, Kruijssen L, Zaal A, O'Toole T, Wiercx L, van Kooyk Y, van Vliet SJ. Disruption of sialic acid metabolism drives tumor growth by augmenting CD8(+) T cell apoptosis. Int J Cancer. 2019:144(9):2290–2302. 10.1002/ijc.32084. 30578646 PMC6519079

[ref18] Crocker PR, Paulson JC, Varki A. Siglecs and their roles in the immune system. Nat Rev Immunol. 2007:7(4):255–266. 10.1038/nri2056. 17380156

[ref19] Edgar LJ, Thompson AJ, Vartabedian VF, Kikuchi C, Woehl JL, Teijaro JR, Paulson JC. Sialic acid ligands of CD28 suppress Costimulation of T cells. ACS Cent Sci. 2021:7(9):1508–1515. 10.1021/acscentsci.1c00525. 34584952 PMC8461770

[ref20] Figueroa MG, Parker LM, Krol K, Zhao M. Distal Lck promoter-driven Cre shows cell type-specific function in innate-like T cells. Immunohorizons. 2021:5(9):772–781. 10.4049/immunohorizons.2100079. 34583938 PMC8612026

[ref21] Fukuda M, Tsuboi S. Mucin-type O-glycans and leukosialin. Biochim Biophys Acta. 1999:1455(2–3):205–217. 10.1016/S0925-4439(99)00067-8. 10571013

[ref22] Garabedian BM, Bashian EE, Wang X, Thompson AJ, Paulson JC. Targeting sialidase to PD1 enhances T cell function and tumor control. ACS Cent Sci. 2025:11(8):1417–1427. 10.1021/acscentsci.5c00510. 40893963 PMC12395300

[ref23] Gillespie W, Paulson JC, Kelm S, Pang M, Baum LG. Regulation of alpha 2,3-sialyltransferase expression correlates with conversion of peanut agglutinin (PNA)+ to PNA- phenotype in developing thymocytes. J Biol Chem. 1993:268(6):3801–3804. 10.1016/S0021-9258(18)53540-7. 8440675

[ref24] Gray MA, Stanczak MA, Mantuano NR, Xiao H, Pijnenborg JFA, Malaker SA, Miller CL, Weidenbacher PA, Tanzo JT, Ahn G, et al. Targeted glycan degradation potentiates the anticancer immune response in vivo. Nat Chem Biol. 2020:16(12):1376–1384. 10.1038/s41589-020-0622-x. 32807964 PMC7727925

[ref25] Hennet T, Chui D, Paulson JC, Marth JD. Immune regulation by the ST6Gal sialyltransferase. Proc Natl Acad Sci U S A. 1998:95(8):4504–4509. 10.1073/pnas.95.8.4504. 9539767 PMC22519

[ref26] Hernandez JD, Nguyen JT, He J, Wang W, Ardman B, Green JM, Fukuda M, Baum LG. Galectin-1 binds different CD43 glycoforms to cluster CD43 and regulate T cell death. J Immunol. 2006:177(8):5328–5336. 10.4049/jimmunol.177.8.5328. 17015718

[ref27] Irons EE, Gc S, Lau JTY. Sialic acid in the regulation of blood cell production, differentiation and turnover. Immunology. 2024:172(4):517–532. 10.1111/imm.13780. 38503445 PMC11223974

[ref28] Izzati FN, Choksi H, Giuliana P, Abd-Rabbo D, Elsaesser H, Blundell A, Affe V, Kannen V, Jame-Chenarboo Z, Schmidt EN, et al. Presentation of immunoregulatory sialoglycans on T cells is divergent between mice and humans. Cell Rep. 2025:44(7):115933. 10.1016/j.celrep.2025.115933. 40608513

[ops-bib-reference-rmsz8s4k2yjacpn9] Kaech SM, Cui W. Transcriptional control of effector and memory CD8+ T cell differentiation. Nat Rev Immunol. 2012;12(11):749‑761. 10.1038/nri3307. PMC4137483 23080391

[ref29] Kao C, Sandau MM, Daniels MA, Jameson SC. The sialyltransferase ST3Gal-I is not required for regulation of CD8-class I MHC binding during T cell development. J Immunol. 2006:176(12):7421–7430. 10.4049/jimmunol.176.12.7421. 16751387

[ref30] Kasahara T, Chang TC, Yoshioka H, Urano S, Egawa Y, Inoue M, Tahara T, Morimoto K, Pradipta AR, Tanaka K. Anticancer approach by targeted activation of a global inhibitor of sialyltransferases with acrolein. Chem Sci. 2024:15(25):9566–9573. 10.1039/D4SC00969J. 38939146 PMC11206204

[ref31] Klein L, Kyewski B, Allen PM, Hogquist KA. Positive and negative selection of the T cell repertoire: what thymocytes see (and don't see). Nat Rev Immunol. 2014:14(6):377–391. 10.1038/nri3667. 24830344 PMC4757912

[ref32] Laubli H, Varki A. Disrupting Siglec-mediated interactions to develop immunotherapies for cancer treatment. Expert Rev Anti-Infect Ther. 2025:29(9):613–619. 10.1080/14728222.2025.2557281. 40899789

[ref33] Leick KM, Pinczewski J, Mauldin IS, Young SJ, Deacon DH, Woods AN, Bosenberg MW, Engelhard VH, Slingluff CL Jr. Patterns of immune-cell infiltration in murine models of melanoma: roles of antigen and tissue site in creating inflamed tumors. Cancer Immunol Immunother. 2019:68(7):1121–1132. 10.1007/s00262-019-02345-5. 31134297 PMC6887106

[ref34] Lin SY, Schmidt EN, Takahashi-Yamashiro K, Macauley MS. Roles for Siglec-glycan interactions in regulating immune cells. Semin Immunol. 2025:77:101925. 10.1016/j.smim.2024.101925. 39706106

[ref35] Luo Z, Ji Y, Tian D, Zhang Y, Chang S, Yang C, Zhou H, Chen ZK. Galectin-7 promotes proliferation and Th1/2 cells polarization toward Th1 in activated CD4+ T cells by inhibiting the TGFbeta/Smad3 pathway. Mol Immunol. 2018:101:80–85. 10.1016/j.molimm.2018.06.003. 29890367

[ref36] Macauley MS, Crocker PR, Paulson JC. Siglec-mediated regulation of immune cell function in disease. Nat Rev Immunol. 2014:14(10):653–666. 10.1038/nri3737. 25234143 PMC4191907

[ref37] Marino JH, Tan C, Davis B, Han ES, Hickey M, Naukam R, Taylor A, Miller KS, Van De Wiele CJ, Teague TK. Disruption of thymopoiesis in ST6Gal I-deficient mice. Glycobiology. 2008:18(9):719–726. 10.1093/glycob/cwn051. 18535087 PMC2733770

[ref38] Moody AM, Chui D, Reche PA, Priatel JJ, Marth JD, Reinherz EL. Developmentally regulated glycosylation of the CD8alphabeta coreceptor stalk modulates ligand binding. Cell. 2001:107(4):501–512. 10.1016/S0092-8674(01)00577-3. 11719190

[ref39] Mosely SI, Prime JE, Sainson RC, Koopmann JO, Wang DY, Greenawalt DM, Ahdesmaki MJ, Leyland R, Mullins S, Pacelli L, et al. Rational selection of syngeneic preclinical tumor models for immunotherapeutic drug discovery. Cancer Immunol Res. 2017:5(1):29–41. 10.1158/2326-6066.CIR-16-0114. 27923825

[ref40] Motran CC, Molinder KM, Liu SD, Poirier F, Miceli MC. Galectin-1 functions as a Th2 cytokine that selectively induces Th1 apoptosis and promotes Th2 function. Eur J Immunol. 2008:38(11):3015–3027. 10.1002/eji.200838295. 18991278 PMC2782404

[ref41] Nan X, Carubelli I, Stamatos NM. Sialidase expression in activated human T lymphocytes influences production of IFN-gamma. J Leukoc Biol. 2007:81(1):284–296. 10.1189/jlb.1105692. 17028199

[ref42] Nguyen JT, Evans DP, Galvan M, Pace KE, Leitenberg D, Bui TN, Baum LG. CD45 modulates galectin-1-induced T cell death: regulation by expression of core 2 O-glycans. J Immunol. 2001:167(10):5697–5707. 10.4049/jimmunol.167.10.5697. 11698442

[ref43] O'Neill A, Zakaria N, Egan H, Treacy O, Hogan AM, O'Dwyer M, Hynes SO, Ryan AE. The tumour Glyco-code: sialylation as a mediator of stromal cell immunosuppression in the tumour microenvironment. Eur J Immunol. 2025:55(7):e70000. 10.1002/eji.70000. 40667828 PMC12265401

[ref44] Oswald DM, Jones MB, Cobb BA. Modulation of hepatocyte sialylation drives spontaneous fatty liver disease and inflammation. Glycobiology. 2020:30(5):346–359. 10.1093/glycob/cwz096. 31742330 PMC7175972

[ref45] Pearce OM, Laubli H. Sialic acids in cancer biology and immunity. Glycobiology. 2016:26(2):111–128. 10.1093/glycob/cwv097. 26518624

[ref46] Pinho SS, Macauley MS, Laubli H. Tumor glyco-immunology, glyco-immune checkpoints and immunotherapy. J Immunother Cancer. 2025:13(6):e012391. 10.1136/jitc-2025-012391. 40533266 PMC12182193

[ref47] Priatel JJ, Chui D, Hiraoka N, Simmons CJ, Richardson KB, Page DM, Fukuda M, Varki NM, Marth JD. The ST3Gal-I sialyltransferase controls CD8+ T lymphocyte homeostasis by modulating O-glycan biosynthesis. Immunity. 2000:12(3):273–283. 10.1016/S1074-7613(00)80180-6. 10755614

[ref48] Ray A, Buon L, Wan X, Du T, Song Y, Pillai SC, Musa MA, Fang T, Wang M, Anderson KC. Targeting Siglec-sialic acid checkpoint to reverse immune dysfunction mediated by plasmacytoid dendritic cells in myeloma. Blood Adv. 2025:9(19):5085–5090. 10.1182/bloodadvances.2025015980. 40311068 PMC12516034

[ref49] Sampson JF, Suryawanshi A, Chen WS, Rabinovich GA, Panjwani N. Galectin-8 promotes regulatory T-cell differentiation by modulating IL-2 and TGFbeta signaling. Immunol Cell Biol. 2016:94(2):213–219. 10.1038/icb.2015.72. 26282995 PMC4747822

[ref50] Schetters STT, Rodriguez E, Kruijssen LJW, Crommentuijn MHW, Boon L, Van den Bossche J, Den Haan JMM, Van Kooyk Y. Monocyte-derived APCs are central to the response of PD1 checkpoint blockade and provide a therapeutic target for combination therapy. J Immunother Cancer. 2020:8(2):e000588. 10.1136/jitc-2020-000588. 32690667 PMC7371367

[ref51] Schmidt M, Linder AT, Korn M, Schellenberg N, Meyer SJ, Nimmerjahn F, Werner A, Abeln M, Gerardy-Schahn R, Munster-Kuhnel AK, et al. Sialic acids on T cells are crucial for their maintenance and survival. Front Immunol. 2024:15:1359494. 10.3389/fimmu.2024.1359494. 38947328 PMC11211268

[ref52] Sierra-Ulloa D, Fernandez J, Cacelin M, Gonzalez-Aguilar GA, Saavedra R, Tenorio EP. alpha2,6 sialylation distinguishes a novel active state in CD4(+) and CD8(+) cells during acute toxoplasma gondii infection. Front Immunol. 2024:15:1429302. 10.3389/fimmu.2024.1429302. 39253089 PMC11381403

[ref53] Stanczak MA, Rodrigues Mantuano N, Kirchhammer N, Sanin DE, Jacob F, Coelho R, Everest-Dass AV, Wang J, Trefny MP, Monaco G, et al. Targeting cancer glycosylation repolarizes tumor-associated macrophages allowing effective immune checkpoint blockade. Sci Transl Med. 2022:14(669):eabj1270. 10.1126/scitranslmed.abj1270. 36322632 PMC9812757

[ref54] Su EW, Bi S, Kane LP. Galectin-9 regulates T helper cell function independently of Tim-3. Glycobiology. 2011:21(10):1258–1265. 10.1093/glycob/cwq214. 21187321 PMC3167474

[ref55] Toscano MA, Bianco GA, Ilarregui JM, Croci DO, Correale J, Hernandez JD, Zwirner NW, Poirier F, Riley EM, Baum LG, et al. Differential glycosylation of TH1, TH2 and TH-17 effector cells selectively regulates susceptibility to cell death. Nat Immunol. 2007:8(8):825–834. 10.1038/ni1482. 17589510

[ref56] Tsai HE, Chen CL, Chang TT, Fu CW, Chen WC, Perez S, Hsiao PW, Tai MH, Li WS. Development of a novel, potent, and selective Sialyltransferase inhibitor for suppressing cancer metastasis. Int J Mol Sci. 2024:25(8):4283(1-17). 10.3390/ijms25084283. PMC11050067 38673867

[ref57] Varki A, Gagneux P. Multifarious roles of sialic acids in immunity. Ann N Y Acad Sci. 2012:1253(1):16–36. 10.1111/j.1749-6632.2012.06517.x. 22524423 PMC3357316

[ref58] Weinreich MA, Hogquist KA. Thymic emigration: when and how T cells leave home. J Immunol. 2008:181(4):2265–2270. 10.4049/jimmunol.181.4.2265. 18684914 PMC2861282

[ref59] Wen RM, Stark JC, Marti GEW, Fan Z, Lyu A, Garcia Marques FJ, Zhang X, Riley NM, Totten SM, Bermudez A, et al. Sialylated glycoproteins suppress immune cell killing by binding to Siglec-7 and Siglec-9 in prostate cancer. J Clin Invest. 2024:134(24):e180282(1-15). 10.1172/JCI180282. PMC11645153 39436703

[ref60] Xiao R, Tian Y, Zhang J, Li N, Qi M, Liu L, Wang J, Li Z, Zhang J, Zhao F, et al. Increased Siglec-9/Siglec-9L interactions on NK cells predict poor HCC prognosis and present a targetable checkpoint for immunotherapy. J Hepatol. 2024:80(5):792–804. 10.1016/j.jhep.2024.01.028. 38331327

[ref61] Yang Z, Hou Y, Grande G, Cho JH, Wang C, Shi Y, Zak J, Wan Y, Qin K, Liu D, et al. Targeted desialylation and cytolysis of tumour cells by fusing a sialidase to a bispecific T-cell engager. Nat Biomed Eng. 2024:8(5):499–512. 10.1038/s41551-024-01202-w. 38693431 PMC11577304

[ref62] Yu X, Qian J, Ding L, Yin S, Zhou L, Zheng S. Galectin-1: a traditionally immunosuppressive protein displays context-dependent capacities. Int J Mol Sci. 2023:24(7):6501(1-28). 10.3390/ijms24076501. PMC10095249 37047471

[ref63] Zhang DJ, Wang Q, Wei J, Baimukanova G, Buchholz F, Stewart AF, Mao X, Killeen N. Selective expression of the Cre recombinase in late-stage thymocytes using the distal promoter of the Lck gene. J Immunol. 2005:174(11):6725–6731. 10.4049/jimmunol.174.11.6725. 15905512

[ref64] Zhang H, Tsui CK, Castillo JG, Kim EJY, Evangelista A, Liu H, Joe LK, Twells N, Robey EA, Mahal LK, et al. Age-related remodeling of the sialoglycans dampens murine CD8(+) T cell function. Sci Adv. 2025:11(39):eadw6755. 10.1126/sciadv.adw6755. 41004592 PMC12467053

[ref65] Zhao D, Liu M, Zeng W, Chen S, Bibi S, Wang M, Huang X, Zhu F, Zheng P, Gao Y, et al. A bispecific antibody targeting the Ig domains of Siglec-E displays enhanced antitumor effects. Int J Biol Macromol. 2024:281(Pt 4):136635. 10.1016/j.ijbiomac.2024.136635. 39419134

