# Supplementary material for: Impact of sialyltransferase deletion in mature T cells on control of subcutaneous and metastatic cancers
Source: Glycobiology. 2026 Aug 19;36(10):cwag065. doi: 10.1093/glycob/cwag065 (PMC13513753; doi:10.1093/glycob/cwag065)
Supplement: Supplementary file 1 [file supplementary_materials_cwag065.zip › supporting document.docx]

**Impact of sialyltransferase deletion in mature T cells on control of subcutaneous and metastatic cancers**

Xiaoshuang Wang^1,2^ and James C. Paulson^2,^*

^1^ The School of Medicine, Nankai University, 94 Weijin Road, Tianjin 300071, China

^2^ Department of Immunology and Microbiology, The Scripps Research Institute, La Jolla, California 92037, United States.

* Corresponding author:

Email: [jpaulson@scripps.edu](mailto:jpaulson@scripps.edu). Address:

Department of Immunology and Microbiology

The Scripps Research Institute

10550 N. Torrey Pines Rd,

La Jolla, CA 92014

**Running head:** Mature T cell sialyltransferases impact tumor growth

**Supplementary data items:** Supplementary Figures S1-S6

**Keywords:** CD8^+^ T cell/metastasis/sialic acid/sialyltransferases/tumor

**Supplementary Figure S1.** Genotyping of T-ST3KO and T-ST6KO mice. **(A)** Schematic diagram of T-ST3KO mice construction. Primers used for genotyping are shown as P1 and P2. **(B)** Gel image of T-ST3KO mouse genotyping. **(C)** Schematic diagram of T-ST6KO mice construction. Primers used for genotyping are shown as P3 and P4. **(D)** Agarose gel image of T-ST6KO mouse genotyping.

**Alt text:** Schematic diagrams of construction and gel images of genotyping for T-ST3KO (panels A and B) and T-ST6KO mice (panels C and D).

**Supplementary Figure S2.** T-ST3KO mice, but not T-ST6KO, display reduced inguinal lymph node T cell abundance and increased apoptosis. ST3Gal1^fl/fl^ and ST6Gal1^fl/fl^ mice were used as the corresponding WT mice for T-ST3KO and T-ST6KO mice, respectively. **(A-F)** Analysis of T-ST3KO inguinal lymph node T cells. **(A)** Flow cytometry histograms of T cells from wild type (WT) and dLck-Cre generated T-ST3KO mice stained with PE-labeled PNA (PNA-PE) gated for CD4^+^ and CD8^+^ T cells. **(B)** Mean fluorescence intensity (MFI) for PNA-PE in panel A. **(C)** Flow cytometry contour plot of CD4^+^ and CD8^+^ T cells. **(D)** Relative proportions of cells from panel C. **(E)** Total number of CD4^+^ and CD8^+^ T cells. **(F)**  MFI of CD4^+^ and CD8^+^ T cells stained with annexin V (PE/Cy7-labeled). **(G-L)** Analysis of T-ST6KO inguinal lymph node T cells. **(G)** Flow cytometry histograms of T cells from WT and dLck-Cre generated T-ST6KO mice stained with SNA-PE gated for CD4^+^ and CD8^+^ T cells. **(H)** MFI for SNA-PE in panel G. **(I)** Flow cytometry contour plot of CD4^+^ and CD8^+^ T cells. **(J)** Relative proportions of cells from panel I. **(K)** Total number of CD4^+^ and CD8^+^ T cells. **(L)**  MFI of CD4^+^ and CD8^+^ T cells stained with annexin V (PE/Cy7-labeled). Cells were gated from live populations and data presented as mean ± SEM. Comparisons were analyzed by multiple unpaired t-tests corrected by the Holm-Šidák method. Differences are considered statistically significant at ns p > 0.05 and * p ≤ 0.05.

**Alt text:** Lectin binding, cell proportions, and annexin V levels of inguinal lymph node T cells from T-ST3KO mice (panels A to F) or T-ST6KO mice (panels A to F) compared with their parental WT T cells.

**Supplementary Figure S3**. CD8^+^ T cells of T-ST3KO mice, but not T-ST6KO mice, display enhanced *in vitro* activation with CD80-Fc. Splenic T cells were isolated and purified from 6-8-week-old T-ST3KO, T-ST6KO, and their parental WT mice. The corresponding WT T cells were treated with or without vibrio cholera (VC) sialidase at 37 °C for 30 minutes. The T-ST3KO, T-ST6KO, and their respective sialidase-treated WT T cells (WT±VC) were then cultured with 250 ug/mL CD80-Fc in an anti-CD3 pre-coated flat-bottom 96 well plate at a density of 0.1 M/well for 48 hours. **(A)** Flow cytometry contour plot of activated (defined as CD25^high^ and/or CD69^high^) WT/T-ST3KO CD8^+^ T cells. **(B)** Relative proportions of cells from panel A. **(C)** Flow cytometry contour plot of activated WT/T-ST6KO CD8^+^ T cells. **(D)** Relative proportions of cells from panel C. **(E)** Flow cytometry histogram of WT/T-ST3KO CD8^+^ T cells stained with BUV737-labeled anti-PD-1. **(F)** Relative proportions of PD-1 positive cells from panel E. **(G)** Flow cytometry histogram of WT/T-ST6KO CD8^+^ T cells stained with BUV737-labeled anti-PD-1. **(H)** Relative proportions of PD-1 positive cells from panel G. **(I)** Flow cytometry histogram of WT/T-ST3KO CD8^+^ T cells stained with APC-labeled anti-IL-2. **(J)** Relative proportions of IL-2 positive cells from panel I. **(K)** Flow cytometry histogram of WT/T-ST6KO CD8^+^ T cells stained with APC-labeled anti-IL-2. **(L)** Relative proportions of IL-2 positive cells from panel K. **(M)** Flow cytometry contour plot of naïve (defined as CD44 negative and CD62L positive) and memory-like (defined as CD44 positive) WT/T-ST3KO CD8^+^ T cells. **(N and O)** Relative proportions of naïve subset (N) and memory-like subset (O) from panel M. **(P)** Flow cytometry contour plot of naïve and memory-like WT/T-ST6KO CD8^+^ T cells. **(Q)** Relative proportions of naïve subset (N) and memory-like subset (O) from panel P. Data are presented as mean ± SEM. Comparisons were analyzed by one-way ANOVA followed by Fisher’s LSD test. Differences are considered statistically significant at ns p > 0.05, * p ≤ 0.05, ** p ≤ 0.01, and *** p ≤ 0.001.

**Alt text:** The proportions of activated, naïve, and memory-like CD8^+^ T cell subsets and the expression of PD-1 and IL-2 in CD8^+^ T cells from T-ST3KO or T-ST6KO mice compared with their own parental WT T cells pre-treated with/without vibrio cholera sialidase. Splenic T cells were activated by anti-CD3 plus CD80-Fc for 48 hours.

**Supplementary Figure S4**. CD4^+^ T cells of both T-ST3KO and T-ST6KO mice display silimar *in vitro* activation compared to their parental WT T cells. Splenic T cells were isolated and purified from 6-8-week-old T-ST3KO, T-ST6KO, and their parental WT mice, and cultured with soluble anti-CD28 in an anti-CD3 pre-coated flat-bottom 96 well plate at a density of 0.1 M/well for 48 hours. ST3Gal1^fl/fl^ and ST6Gal1^fl/fl^ mice were used as the corresponding WT mice for T-ST3KO and T-ST6KO mice, respectively. **(A and B)** Relative proportions of activated (defined as CD25^high^ and/or CD69^high^) WT/T-ST3KO (A) and WT/T-ST6KO (B) CD4^+^ T cells. **(C and D)** Relative proportions of PD-1 positive WT/T-ST3KO (C) and WT/T-ST6KO (D) CD4^+^ T cells. **(E and F)** Relative proportions of IL-2 positive WT/T-ST3KO (E) and WT/T-ST6KO (F) CD4^+^ T cells. **(G and H)** Relative proportions of naïve subset (defined as CD44 negative and CD62L positive) (G) and memory-like subset (defined as CD44 positive) (H) from WT/T-ST3KO CD4^+^ T cells. **(I and J)** Relative proportions of naïve subset (I) and memory-like subset (J) from WT/T-ST3KO CD4^+^ T cells. Data are presented as mean ± SEM. Comparisons were analyzed by unpaired two-tailed Student’s t-tests. Differences are considered statistically significant at ns p > 0.05.

**Alt text:** The similar proportions of activated, naïve, and memory-like CD4^+^ T cell subsets and the similar expression of PD-1 and IL-2 in CD4^+^ T cells from T-ST3KO or T-ST6KO mice compared with their own parental WT T cells. Splenic T cells were activated by anti-CD3 plus soluble anti-CD28 for 48 hours.

**Supplementary Figure S5.** T-ST3KO splenic CD8^+^ T cells exhibit enhanced CD80-Fc binding to CD28. Splenic T cells were isolated and purified from 6-8-week-old mice and stained with cell surface markers and mouse CD80-Fc. ST3Gal1^fl/fl^ and ST6Gal1^fl/fl^ mice were used as the corresponding WT mice for T-ST3KO and T-ST6KO mice, respectively. **(A-B)** Delta change in mean fluorescence intensity (MFI) of PE-labeled mouse IgG1 staining in WT and T-ST3KO (A) or T-ST6KO (B) mice. Data are presented as mean ± SEM. Comparisons were analyzed using unpaired two-tailed Student’s t-tests. Differences are considered statistically significant at ns p > 0.05 and ** p ≤ 0.01.

**Alt text:** The binding strength of CD80-Fc to T-ST3KO or T-ST6KO splenic CD8^+^ T cells compared with their parental WT T cells.

**Supplementary Figure S6.** B16F10 tumors grow similarly in T-ST3KO and T-ST6KO mice. 0.5 million B16F10 single-cell suspensions were subcutaneously injected in the right leg flank of 6-8-week-old male T-ST3KO **(A-F)** or T-ST6KO **(G-L)** or their parental WT mice on Day 0. Tumor size was subsequently monitored, and the number of infiltrated immune cells (TILs) was quantified in each tumor at the endpoint. **(A-B)** Summary (A) and individual (B) tumor growth curves of B16F10 tumor-bearing T-ST3KO mice. **(C)** Kaplan-Meier survival analysis. **(D)** Tumor-infiltrating CD45^+^ leukocytes (TILs) total number. **(E-F)** The proportions (E) and the total number (F) of tumor-infiltrating CD4^+^ T cells and CD8^+^ T cells. **(G-H)** Summary (G) and individual (H) tumor growth curve of B16F10 tumor-bearing T-ST6KO mice. **(I)** Kaplan-Meier survival analysis. **(J)** TILs total number. **(K-L)** The proportions (K) and the total number (L) of tumor-infiltrating CD4^+^ T cells and CD8^+^ T cells. Data are presented as mean ± SEM. The survival curves were obtained using the Kaplan-Meier method and compared statistically using the Log-rank (Mantel-Cox) test. Comparisons were analyzed by unpaired two-tailed Student’s t-tests for panels D and J, while by multiple unpaired t-tests corrected by the Holm-Šidák method for other panels. Differences are considered statistically significant at ns p > 0.05 and ** p ≤ 0.01.

**Alt text:** Tumor growth, mouse survival, and the numbers of tumor-infiltrating CD45⁺ leukocytes in B16F10 subcutaneous tumor-bearing T-ST3KO mice (panels A to F) or T-ST6KO mice (panels G to L) compared with their parental WT mice. B16F10 tumors grow similarly in T-ST3KO and T-ST6KO mice.
